# Adsorption of Per- and Polyfluoroalkyl Substances by Edible Nutraceutical-Amended Montmorillonite Clays: In Vitro, In Vivo and In Silico Enterosorption Strategies

**DOI:** 10.1007/s11270-025-07930-2

**Published:** 2025-04-04

**Authors:** Johnson O. Oladele, Xenophon Xenophontos, Meichen Wang, Phanourios Tamamis, Timothy D. Phillips

**Affiliations:** 1 Interdisciplinary Faculty of Toxicology, Texas A&M University, College Station, TX 77843 USA; 2 Department of Veterinary Physiology and Pharmacology, College of Veterinary Medicine & Biomedical Sciences, Texas a&M University, College Station, TX 77843 USA; 3 Artie Mcferrin Department of Chemical Engineering, College of Engineering, Texas A&M University, College Station, TX 77843 USA; 4 Department of Materials Science and Engineering, College of Engineering, Texas A&M University, College Station, TX 77840 USA; 5 Department of Environmental Health Sciences, University of Massachusetts Amherst, Amherst, Massachusetts 01003 USA

**Keywords:** Emerging contaminants, PFAS, Adsorption, Enterosorption, Polyfluoroalkyl substances, Molecular dynamics simulations, Detoxification, Edible clay

## Abstract

Exposure of animals and humans to PFAS through contaminated water and foods pose significant threats to public health. To tackle this challenge, this study aimed to develop edible clays that might enhance the binding, detoxification, and elimination of PFAS in the gastrointestinal tract. Montmorillonite clays (CM) were amended with caffeine (CMCAF), curcumin (CMCUR), and riboflavin (CMRIB), and the binding efficacy for a mixture of four PFAS (PFOS, GenX, PFOA and PFBS) was determined. In vitro studies were used to explore adsorption isotherms while computational simulations investigate PFAS mixture, delineate the contribution of each PFAS molecule to clays and determine if amended clays can contribute to enhanced binding of different PFAS in the mixture. In vivo models (*Lemna minor* and *Hydra vulgaris*) were used to validate in vitro and in silico studies and establish the safety and effectiveness of these amended clays. The resulting Q_max_ and K_d_ values along with the curved shape of the Langmuir plot indicated saturable binding of GenX, PFOA and PFOS to active surfaces of CM and the amended clays. All three clays demonstrated a slightly higher binding capacity for GenX than the parent clay. Furthermore, the simulations elucidated the binding contribution of each PFAS molecule to parent and amended clays as well as predicting how amended clays can contribute to mechanisms of binding of different PFAS in the mixture. The proof-of-concept for the efficacy of the clays was established in *Caenorhabditis elegans, Lemna minor* and *Hydra vulgaris*, where the clays (at 1% w/v inclusion) protected against toxicities of the four PFAS controls. This protection could be attributed to PFAS binding to the amended clays and the biological activities of these nutraceuticals (caffeine, riboflavin, and curcumin) including antioxidative, anti-inflammatory and modulatory activities which mitigate the oxidative stress and inflammatory effects of PFAS. These edible toxin binders may be delivered in mixtures as additives in flavored drinking water and food to decrease PFAS exposure.

**Supplementary Information:**

The online version contains supplementary material available at 10.1007/s11270-025-07930-2.

## Introduction

Per- and polyfluoroalkyl substances (PFAS), also known as "forever chemicals," are a class of highly fluorinated chemicals that have been extensively prepared and broadly used in a variety of products for consumers and commercial processes since the 1940s. They serve as flame retardants, surfactants, additives, lubricants, and pesticides and are produced as byproducts, residues, and intermediates in a variety of techniques (Kissa, 2001; Buck et al., 2011; OEDC, 2018). The most common ways that people are exposed to these chemicals are through using products that contain PFAS, inhaling air that contains PFAS, or ingesting food or water that has been contaminated with PFAS. PFAS pervasiveness and resistance to degradation stems from their many carbon–fluorine (C–F) linkages, and improved thermal and chemical stability (Cousins et al., 2020). In addition, they have the ability to bioaccumulate and sorb due to their lipophobic and hydrophobic properties, and can travel through a variety of environmental pathways, all of which can be harmful to living organisms (Ahrens & Bundschuh, 2014).

PFAS contamination sites are still being found around the world as a result of their extensive use as essential ingredients in aqueous film-forming foams (AFFF), especially around airports and military locations where AFFF is often employed for training and firefighting operations (Kissa, 2001; Sunderland et al., 2019). PFAS coexist alongside microplastics and other additives and polymers, which means they are closely linked to the worldwide problem of plastic pollution (Joo et al., 2021). When fluoropolymers like polyvinyl fluoride (PVF) and polytetrafluorethylene (PTFE) break down, polymeric PFAS can occasionally appear as microplastics (Lohmann et al., 2020). Furthermore, PFAS are frequently used as coatings on synthetic fabrics and plastic components. During the manufacturing, utilization, and disposing processes, these coatings might break down into macro-, meso-, or microplastics based on fibers or particles. Moreover, during production, non-PFAS microplastics like polyvinyl chloride (PVC) may come into contact with PFAS. Several plastics, paper, and textile goods include side-chain fluorinated polymer groups that can emit perfluoroalkyl substances (PFAS) (Washington et al., 2015).

One of the most widely used PFAS in a variety of applications is perfluorooctanoic acid (PFOA), followed by perfluorooctane sulfonate (PFOS). Even though their usage has been discontinued worldwide, the Stockholm Convention on Persistent Organic Chemicals lists them because they are still present in the environment (SSC, 2019). Research on alternatives such as 6:2 Cl-PFAES (6:2 chlorinated polyfluoroalkyl ether sulfonic acid) commercially known as F-53B, GenX (hexafluoropropylene oxide dimer acid or HFPO-DA), perfluorohexane sulfonic acid (PFHxS), and perfluorohexanoic acid (PFHxA), which has attracted much attention, has been spurred by the ongoing concerns regarding PFOA and PFOS (Brase et al., 2021; Gebbink & van Leeuwen, 2020; He et al., 2022; Li et al., 2023). Furthermore, PFOA has been identified by the International Agency for Research on Cancer (IARC) as "possibly carcinogenic to humans" (IARC, 2017). Strong links have been found by epidemiological research between PFOA and PFOS and kidney and testicular cancer; nevertheless, long-term follow-up studies with sizable cohorts and significant exposure contrasts are necessary to draw firm conclusions (Steenland & Winquist, 2021). It's also critical to keep in mind that the toxicity of each PFAS may vary among its isomers (Yu et al., 2015).

Numerous harmful effects are displayed by PFAS, such as neurotoxicity, cytotoxicity, immunotoxicity, reproductive toxicity, hepatotoxicity, carcinogenicity, genotoxicity, hormonal toxicity, and developmental toxicity (Fenton et al., 2021). They have mostly been linked to increased cancer risks, immunological reactions, metabolic disorders, developmental problems, and reproductive effects (Sunderland et al., 2019). PFAS are categorized as possible endocrine disruptors because of their capacity to interfere with nuclear receptors and cause disruptions to the endocrine system (Fenton et al., 2021; Wee & Aris, 2017). Only a small portion of PFAS is presently monitored and regulated, despite the fact that they are suspected environmental pollutants and endocrine disruptors (ITRC, 2022, Sunderland et al., 2019). About 97% of Americans have been reported to have PFAS in their blood, according to research by the Centers for Disease Control and Prevention that used data from the National Health and Nutrition Examination Survey (NHANES) (Lewis et al., 2015). Nevertheless, there are currently no known methods for mitigating unintentional PFAS exposures through food items and drinking water. Thus, there is an urgent need for viable sorbent strategies to (i) remove PFAS in food items and drinking water and (ii) eradicate PFAS compounds in contaminated sites and environments.

Adsorption has been reported as a promising method for PFAS removal due to its efficacy and ease of application (Zhang et al., 2019, 2022; Zhang & Liang 2022). Many traditional and novel adsorbents including composite materials, synthetic polymers, modified minerals, ion exchange resins, and activated carbons have been produced and used to remove PFAS (Boyer et al., 2021; Dixit et al., 2021; Kumar et al., 2020; Sörengård, et al., 2020; Zhang et al., 2011). Remarkably, amended clays such as organoclay have gained popularity for remediation of PFAS and other environmental pollutants due to their low cost, simplicity of production, compatibility with natural soils/sediments, and sustainability of clay minerals (Wang et al., 2021). The current research focus on PFAS adsorption by clays includes enhancement of hydrophobic and electrostatic interactions between clay with PFAS. Recent studies have demonstrated improved adsorption capacity clay for PFAS removal through various amendments and modifications. These include amendment with cationic organic compounds such as choline, chlorophyll, carnitine, chlorophyllin, polyfluoroalkyl quaternary ammonium, hexadecyltrimethylammonium, imidazolium-type ionic liquids, etc. and the resulting organoclays demonstrated improved PFAS adsorption capacity and fast adsorption kinetics (Wang et al., 2021; Zhao et al., 2014; Zhou et al., 2010). Similarly, in vivo studies have been conducted to assess the toxicity of PFAS exposure as well as the effectiveness of the amended clays in reducing PFAS exposure. For instance, recent study from our group utilized the *Lemna minor* bioassay to demonstrate that the inclusion of nutrient-amended clays at 0.1% reduced PFOA and PFOS toxicity (Wang et al., 2021).

Our recent computational and experimental studies predicted and validated that caffeine (CMCAF), curcumin (CMCUR) and riboflavin (CMRIB)-amendments of calcium montmorillonite clay enhanced the binding and sorption of four PFAS at acidic conditions (pH 2) (manuscript under review). This current study investigates detoxification efficacy of the nutraceuticals-amended clay in living organisms. Since studies have shown that carbon-chain classification is vital in PFAS environmental behavior and potential health risk, this study includes a mixture of four PFAS, including long-chain PFOS (C8) and PFOA (C8) and short-chain PFBS (C4) and GenX (C6), respectively. Similarly, PFOS and PFOA are representatives of the most prevalent PFAS in the environment (surface water and biota) and food samples (drinking water, fish and seafood) (Dimitrakopoulou et al., 2024). As a follow-up to our recent studies, we focused on investigating the binding properties of a PFAS mixture using (i) in vitro isothermal analysis of edible nutraceutical-clays, (ii) in vivo study of PFAS in three living organisms, and (iii) in silico computer molecular dynamics (MD) simulations.

## Materials and Methods

### Chemicals and Materials

PFAS (PFBS, GenX, PFOS, and PFOA) analytical standards were procured from Sigma Aldrich (St. Louis, MO, USA). Buffers (pH 4.0, 7.0, and 10.0) and high-performance liquid chromatography (HPLC)-grade acetonitrile were obtained from VWR (Atlanta, GA, USA). Parent montmorillonite, obtained from BASF (Lampertheim, Germany), had the generic formula (Na,Ca)_0.3_(Al,Mg)_2_Si_4_O_10_(OH)_2_•nH_2_O, an external surface area of 70 m^2^/g, a total surface area of approximately 850 m^2^/g, a zeta potential of −31 mV, and a cation exchange capacity of 97 cmol/kg (Kumar et al., 2020). Using previously reported protocols for amendment of clays, nutraceutical-amended clays were created by interposing curcumin, riboflavin, or caffeine between interlayer surfaces with 100% cation exchange capacity in neutral and acidic environments (Wang et al., 2017).

### Chemical Analysis

PFAS analysis was carried out using the protocol reported in our previous studies (Wang et al., 2021) with slight modification. The analysis of the four PFAS compounds (PFBS, GenX, PFOS, and PFOA) was conducted using a Waters Acquity ultraperformance liquid chromatography/tandem mass spectrometer (LC/MS–MS) fitted with a triple quadrupole. The detection and quantification were carried out using an Acquity BEH C18 column (2.1 × 50 mm, 1.7 μm), which was maintained at 40 °C in the column oven. At a flow rate of 0.30 mL/min, a gradient elution was performed for 13 min using 20 mM ammonium acetate (eluent A) and acetonitrile (eluent B). For every analysis, the injection volume was 50 μL. The mass spectrometer was operated with an electrospray ionization interface (ESI) set in a negative ion mode and the spray voltage was kept at 4.5 kV. The source temperature was kept at 450 °C. The monitored precursor and product ions (m/z) for PFBS, GenX, PFOS, and PFOA were 298.9 to 80, 285 to 168.9, 499 to 80, and 413 to 369, respectively. The cone voltage (kV) for PFBS, GenX, PFOS, and PFOA was 35, 20, 40, and 25, respectively. The mass spectrometer was operated under multiple reaction monitoring (MRM) mode. The nebulizer and heater gases were argon gas, whereas the collision and curtain gases were nitrogen gas. Empower analyst software was used to control the LC/MS–MS system and acquire data.

### Sorption Isotherms

Individual PFAS solutions, containing PFBS, GenX, PFOS, and PFOA, were prepared from pure crystals at a concentration of 10 µg/mL in pH 7 ultrapure water, which is the average pH of the intestine. Then, each parent and amended clay at 0.0005% was applied to a concentration gradient ranging from 5 to 100% of 1 mL of PFAS solution. One milliliter of clay suspension, PFAS solution, or blank solution (pH 7 water) were used as controls. A 2 h vibration was applied to all samples using an IKA® electric shaker (VIBRAX VXR basic, Werke, Germany) set to 1000 rpm and 37 °C. After centrifuging the sorbent/PFAS complex for 20 min at 2000 g, the supernatant was analyzed using LC/MS–MS.

### Data Calculations, Analysis and Curve Fitting

The amount of free PFAS in the solution was determined using the PFAS measured by the LC/MS–MS method. The amount of bound PFAS in the adsorption investigation was determined using the concentration difference between the test and control groups, and it was plotted as mol/kg on the isotherms. Plotting these data and determining the values for the variable parameters were done using Table-Curve 2D. Based on the equation that fit the data with the highest correlation coefficients and the randomness of the residuals from triplicate studies, the adsorption isotherm was shown using well-known Langmuir or Freundlich models (Grant and Phillips, 1998). Monolayer adsorption onto a surface with a finite number of identical sites and homogeneous adsorption energies is described by the Langmuir isotherm. The Langmuir equation and functions:

1 $$Langmuir\;model\;q=Q_{max}\frac{{\mathrm{K}}_\mathrm{d}{\mathrm{C}}_\mathrm{w}}{1+{\mathrm{K}}_\mathrm{d}{\mathrm{C}}_\mathrm{w}}$$

where* C*_w_ = equilibrium concentration of OTA (mol L^−1^), *K*_d_ = Langmuir distribution constant, *Q*_max_ = maximum binding capacity (mol kg^−1^), and *q* = the amount of OTA adsorbed (mol kg^−1^).

The Freundlich isotherm is used to describe the adsorption characteristics for a heterogeneous surface. The Freundlich model is represented by the following equation:

2 $$Freundlich\;model\;q=K_fC_w^{1/n}$$

Kf = Freundlich distribution constant, 1/n = degree of heterogenicity.

The free energy (ΔG°) was determined by using the Gibbs free energy equation together with the adsorption parameters as given below:

3 $$\Delta G = \Delta {G}^{^\circ }+RTInK{e}^{^\circ }$$

To determine *Ke°* from *K*_*d*_, the following equation was used:

4 $$K{e}^{^\circ }=\frac{{\mathrm{K}}_{\mathrm{d}} {[\mathrm{adsorbate}]}^{\mathrm{o}}}{\upgamma }$$

where *K*_*e*_*°* is the thermodynamic equilibrium constant, *[adsorbate]°* is the standard concentration of the adsorbate = 1 mol/L, and *γ* is the coefficient of activity, *T* (absolute temperature) = 273 + t (°C) and *R* (gas constant) = 8.314 J/mol/K. When *ΔG* is positive, the adsorption process is not favored and could not be significant (Ke° < 1). However, if the value is negative, it is an indication that adsorption process is enhanced thermodynamically and is proceeding forward (Ke° > 1). *ΔG* will be zero for an adsorption system in equilibrium.

### Computational Methods

We used molecular dynamics (MD) simulations to investigate the binding properties of PFAS mixtures in complex with parent (unamended) and nutraceutical-amended clays. PFAS mixtures were comprised of GenX, PFOA, PFOS and PFBS. The simulations were performed using INTERFACE FF (Heinz et al., 2013) force field for clays, and CGenFF (Vanommeslaeghe & MacKerell, 2012) for all the rest. The protonation state of PFAS mixtures and amending compounds is provided in Table S1. Simulation setup was performed analogously as in previous studies of our lab, enabled by CHARMM-GUI (Brooks et al., 2009; Jo et al., 2008; Lee et al., 2016), with particular adjustments introduced. Setup was performed in CHARMM (Brooks et al., 2009), and simulations were performed using OpenMM (Eastman et al., 2017). Upon completion of simulations, we performed analysis using in-house programs, investigating the binding percentage probabilities of PFAS molecules to parent and amended clays, and delineating key mechanisms of binding, by decomposing PFAS and the amendments into chemical groups as shown in Figures S1 and S2, respectively. Interactions between PFAS molecules with parent or nutraceutical-amended clays were decomposed into different types. Detailed description of the methods and analysis is provided in the Supporting Information.

### In Vivo Study with* Hydra vulgaris*

*Hydra vulgaris* is a recognized in vivo model for examining the toxicity of PFAS (Wang et al., 2021). Environmental Canada in Montreal supplied the *Hydra vulgaris* organisms used in this study, which were bred at a consistent temperature of 18 °C in hydra medium that contained 4 mg/L EDTA, 115 mg/L N-tris[hydroxymethyl]methyl-2-aminoethanesulfonic acid (TES), and 147 mg/L CaCl_2_ in 18.2 MΩ water that was adjusted to pH 6.9–7.0. Using an established hydra classification scoring system ranging from 0 to 10, hydra morphology serves as a biomarker for estimating the toxicity of chemicals under a dissecting microscope. This system assigns a score of 0 to a hydra that is thought to be dead or dissolved, and a score of 10 to one that is healthy and has long tentacles. Hydra were treated at different PFAS concentrations ranging from 2.5 to 100 µg/mL for 92 h in order to investigate the toxicity profile of PFAS. Detoxification potentials of the amended clays were evaluated by treating 25 µg/mL of PFAS with 1% (w/v) of the parent clay and the amended clays in hydra media and then exposure to the hydra colony for 92 h. Two hydra colonies each in 2 mL of testing media at 18 °C made up each of the experimental groups.

### In Vivo Study with* Lemna minor*

Previous studies have established *Lemna minor* as a good model for the study of PFAS and other emerging environmental toxic chemicals. In this study, *Lemna minor* was grown in a well-controlled setting after being purchased from AquaHabit in England. Using a standardized procedure, a Steinberg nutritional medium was produced to assist the plant's growth which contained 0.41 mM MgSO_4_, 0.072 mM K2HPO_4_, 0.66 mM KH_2_PO_4_, 1.25 mM Ca(NO_3_)_2_, 3.46 mM KNO_3_, 2.81 μM FeCl_3_, 0.91 μM MnCl_2_, 0.18 μM Na_2_MoO_4_, 0.63 μM ZnSO_4_, 1.94 μM H_3_BO_3_, 4.03 μM EDTA; at pH 5.5 ± 0.2 (Drost et al., 2007). Cool white fluorescent lamps kept at 400 ft-c intensity and with an average temperature of 25 °C were used to provide light for *Lemna minor*. Two randomly selected colonies of *Lemna minor*, each with three fronds, were place into 24 well plates. After that, they were incubated for seven days with the lids slightly unfastened. The plants were exposed to a range of PFAS concentrations for the toxicity analysis from 2.5 to 40 µg/mL. A dose-dependent detoxification evaluation was conducted using 1% (w/v) inclusion of both the parent clay and three amended clays with 40 µg/mL of PFAS for 7 days. With NIH's Image J software (Bethesda, MD), the experiment was monitored every day for variations in surface area and frond number. To extract chlorophyll, *Lemna minor* were homogenized in 1.5 mL of 80% acetone on the final day of the study. A UV–Vis scanning spectrophotometer (Shimadzu UV-1800, Kyoto, Japan) was used to measure the amount of chlorophyll in each homogenate after incubation for 48 h at −4 °C.

### In Vivo Study with *Caenorhabditis elegans*

This study used nematodes as another in vivo model to investigate the toxicity of PFAS and the protective effects of amended clays. According to Wang et al. (2021), *Caenorhabditis elegans* are well-established as a sensitive organism for assessing PFAS toxicity. The Caenorhabditis Genetics Centre (CGC, University of Minnesota) provided wildtype (Bristol N2) *C. elegans* and the *E. coli* strains NA22 and OP50-1. The worms were synchronized using the approach described by Wang et al. (2021). In brief, the nematodes were maintained at 20 °C and cultivated on 8P media that included 8 × 10^8^ cells/mL of *E. coli* NA22. Age-synchronized L1 nematodes were created by bleaching and washing. Egg culture solutions were shaken at 2.5 rpm on a rocking platform (VWR, Radnor, PA) in the dark for eighteen h at 20 °C.

The detoxification ability of the amended clays against PFAS induced toxic effects on *Caenorhabditis elegans* was examined after the incubation period. After counting the worms, they were put in an Eppendorf tube and exposed to supernatant obtained from the reaction complex of each of the amended clays and 50 µg/mL of PFAS. The tube was filled with the appropriate amount of K media and *E. coli* OP50-1 at 20 °C. It was then shaken at 2.5 rpm on a rocking platform in the dark for 24 h. The survival rate of the worms was calculated by counting the number of worms that were pulled using a coated 10 µl pipette. Four nematodes per group were evaluated for motility using the nose contact technique under an Olympus SZ61 zoom stereomicroscope (Olympus, Waltham, MA). After being separated, the nematodes were put on a brand new agar plate and allowed to develop for 24 h at 20 °C. The CellSens Entry (standard version 3) program (Olympus, Waltham, MA) was used to measure each nematode's body length after they had been paralyzed with 25 mM sodium azide. The relative body lengths were calculated as a proportion of the medium control group to adjust to 100%.

## Results

### Adsorption of PFBS, PFOS, GenX and PFOA onto Amended Clays at pH 7

**Fig. 1:**
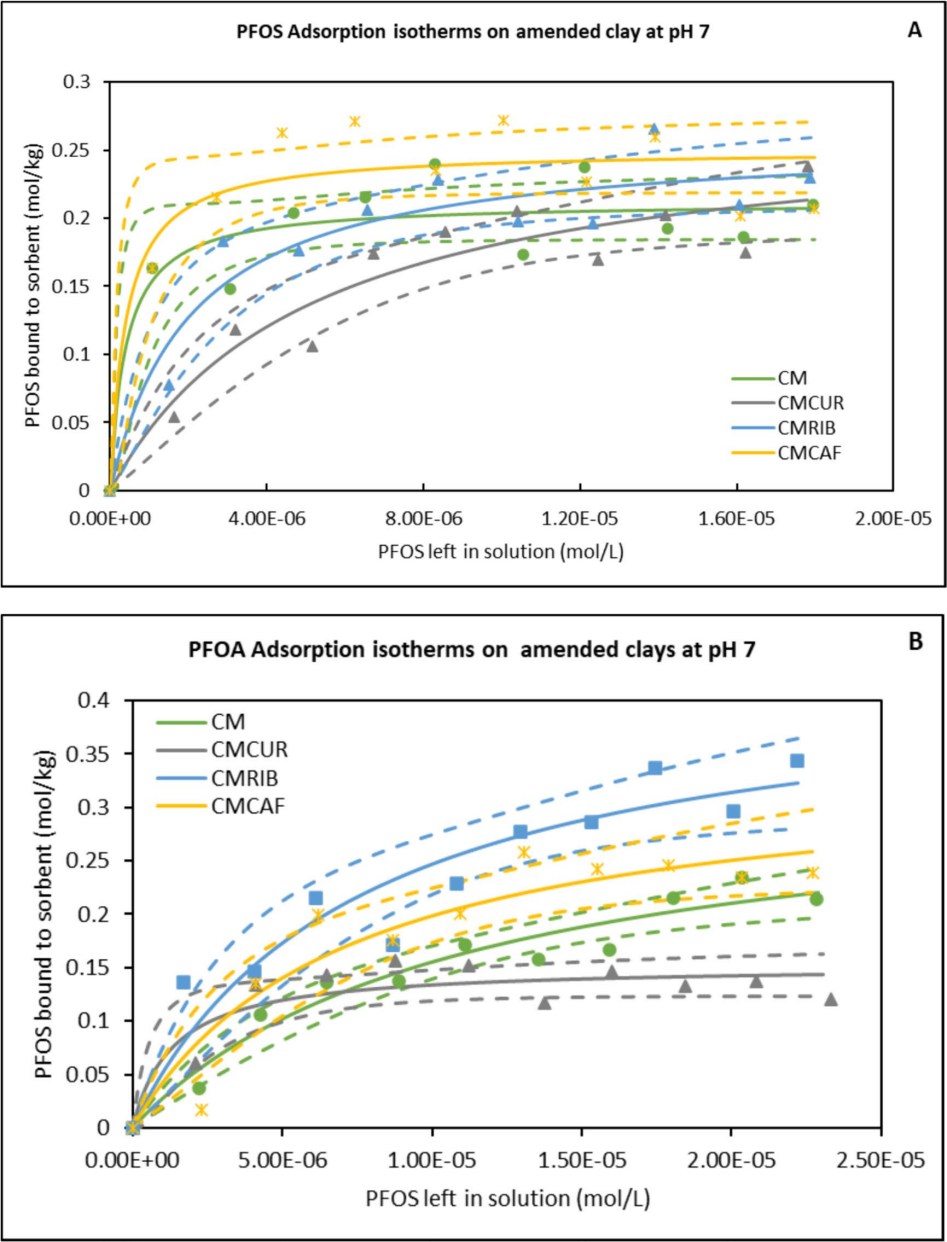

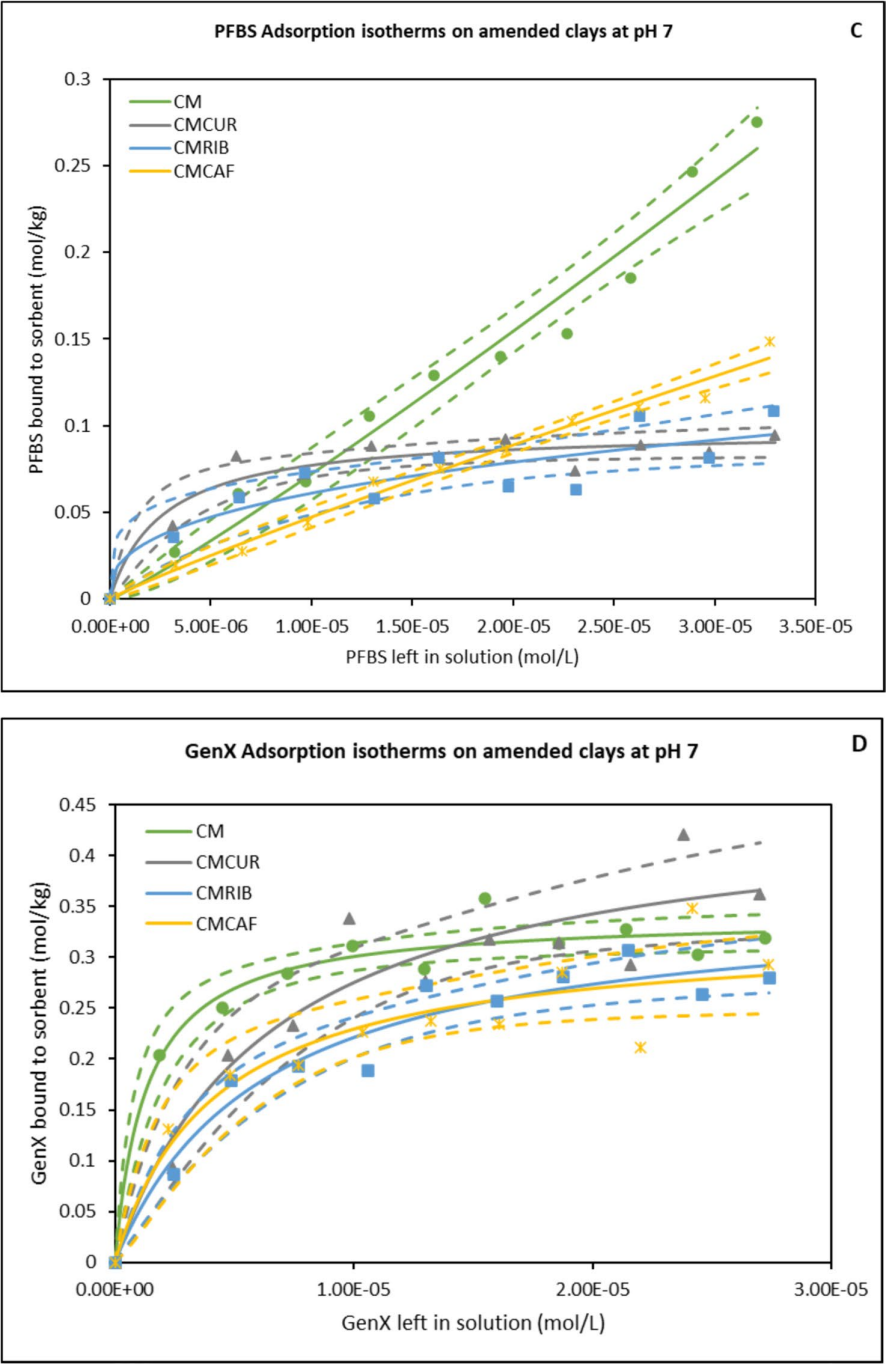
Adsorption isotherms of PFAS onto binding surfaces of the amended clays (**A**) PFOS (**B**) PFOA (**C**) PFBS (**D**) GenX at pH7. CM: Calcium montmorillonite; CMCUR: Curcumin-amended Calcium montmorillonite; CMRIB; Riboflavin-amended Calcium montmorillonite; CMCAF: Caffeine-amended Calcium montmorillonite

To replicate intestinal neutral pH conditions, adsorption isotherms for the four PFAS (PFOS, PFOA, PFBS, and GenX) used in this investigation were carried out at pH 7 (Fig. 1A-D). At 37 °C, all of the PFBS isotherms, with the exception of CMCUR, fit the Freundlich model. All PFOS, PFOA, and GenX isotherms fit the Langmuir models. For PFAS binding to the parent clay (CM) and the amended clays (CMCUR, CMCAF, and CMRIB), these models were used to calculate the degree of heterogenicity (1/n), binding affinity (Kd), and binding capacity (Qmax) values (Table 1). The curved Langmuir plots and the associated Qmax and Kd values showed that GenX, PFOA, and PFOS bound saturably to the active surfaces of the amended clays and parent clay (Fig. 1). The amended clays, CMCAF, CMCUR and CMRIB had slightly higher binding capacity for PFOS than CM, with Qmax values equal to 0.25 mol/kg, 0.27 mol/kg and 0.26 versus 0.21 mol/kg, respectively. However, all bound with high affinity as shown by binding affinities in the range of 10^5^ to 10^6^ (Table 1).

**Table 1:** Parameters and correlation coefficients of adsorption at pH 7

		**PFOS**		
	**Q**_**max**_	**K**_**d**_	**∆G**	**r**^**2**^
CM	0.21	2.47 × 10^6^	−24.16	0.86
CMCAF	0.25	2.47 × 10^6^	−24.11	0.87
CMCUR	0.27	1.94 × 10^5^	−24.49	0.91
CMRIB	0.26	4.81 × 10^5^	−24.40	0.90
		**PFOA**		
CM	0.33	9.04 × 10^4^	−23.58	0.95
CMCAF	0.34	1.40 × 10^5^	−23.88	0.90
CMCUR	0.15	7.31 × 10^5^	−22.06	0.83
CMRIB	0.43	1.33 × 10^5^	−24.88	0.91
		**GenX**		
CM	0.34	7.56 × 10^5^	−24.16	0.97
CMCAF	0.33	2.38 × 10^5^	−23.93	0.87
CMCUR	0.45	1.54 × 10^5^	−24.50	0.91
CMRIB	0.36	1.61 × 10^5^	−23.80	0.95
		**PFBS**		
CMCUR	0.10	3.78 × 10^5^	−20.52	0.92
	**1/n**	**K**_**f**_	**∆G**	**r**^**2**^
CM	0.91	2.24 × 10^4^	−23.35	0.97
CMCAF	1.10	1.66 × 10^3^	−21.71	0.99
CMRIB	2.71	4.32 × 10^1^	−20.88	0.83

Q_max_: Binding capacity (mol/kg); r^2^: Correlation coefficient; ∆G: Gibbs free energy (kJ/mol); K_d_: binding affinity; K_f_: Freundlich distribution constant; 1/n: Degree of heterogenicity

CMCUR and CMRIB also showed a greater ability to bind PFOA than the parent clay. CMCAF and CMRIB had Qmax values of 0.34 and 0.43 mol/kg respectively, while CM had a Qmax of 0.33 mol/kg. The results showed that adding caffeine and riboflavin to CM only slightly increased its ability to bind PFOA. The binding capability of CMCUR, and CMRIB for GenX was higher than that of the parent clay (0.34 mol/kg). Their Qmax values were 0.45 mol/kg and 0.36 mol/kg, respectively. CMCAF was not different from the control. The values of Gibbs free energy of the reactions between each PFAS and clays ranged between -/20.52/ kJ/mol and -/24.88/ kJ/mol (Table 1).

### Computational Results

**Fig. 2:**
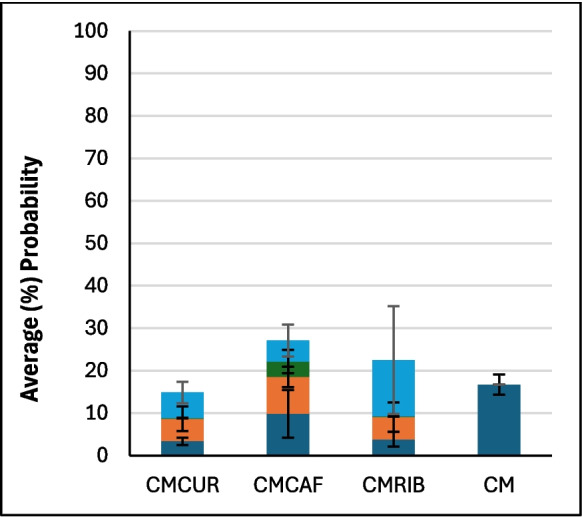
The average percentage binding probability of PFAS mixtures (6 GenX molecules, 6 PFOA molecules, 6 PFOS molecules, and 6 PFBS molecules), to different supplement-amended clays. In each case, the average percentage binding probability of PFAS mixtures to the parent clay (CM) is also shown. Direct, direct-assisted, direct-helped, and indirect-assisted interactions are shown in dark blue, orange, green, and cyan, respectively. The average values are calculated from triplicate runs. Error bars denote standard deviation values calculated from triplicate runs

**Fig. 3:**
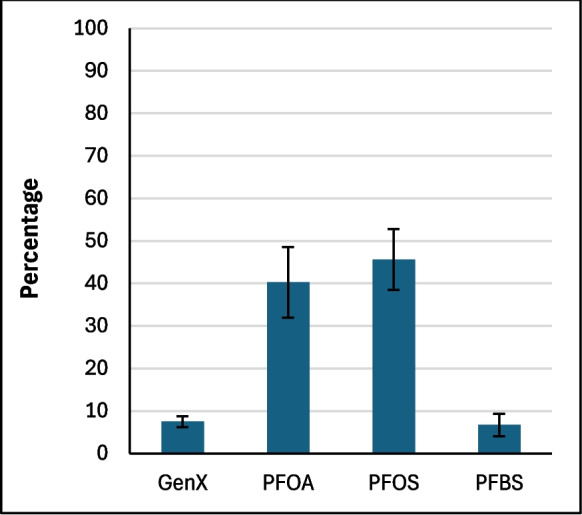
The average percentage decomposition of the binding of PFAS mixtures to parent clay (CM), with respect to the different PFAS species (mixtures comprised 6 GenX molecules, 6 PFOA molecules, 6 PFOS molecules, and 6 PFBS molecules). The average values are calculated from triplicate runs. Error bars denote standard deviation values calculated from triplicate runs

**Fig. 4:**
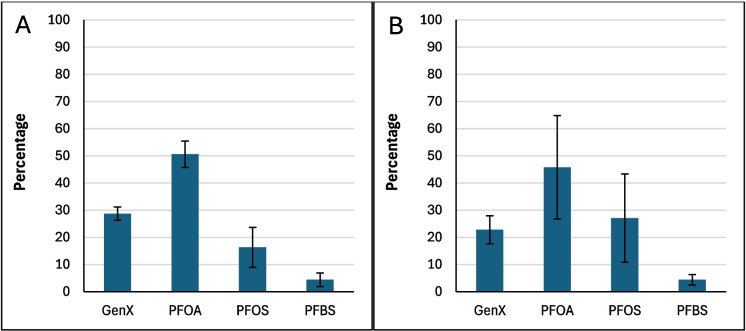
Panels A and B show the average percentage decomposition of the interactions of PFAS mixtures with the clay and the amendments of CMCAF, respectively, with respect to the different PFAS species. Mixtures comprised 6 GenX molecules, 6 PFOA molecules, 6 PFOS molecules, and 6 PFBS molecules. The average values are calculated from triplicate runs. Error bars denote standard deviation values calculated from triplicate runs

**Fig. 5:**
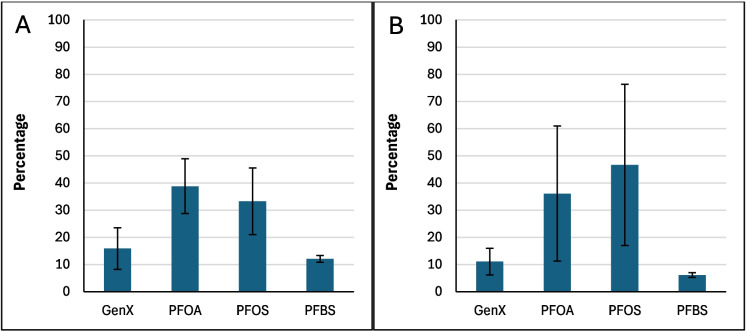
Panels A and B show the average percentage decomposition of the interactions of PFAS mixtures with the clay and the amendments of CMCUR, respectively, with respect to the different PFAS species. Mixtures comprised 6 GenX molecules, 6 PFOA molecules, 6 PFOS molecules, and 6 PFBS molecules. The average values are calculated from triplicate runs. Error bars denote standard deviation values calculated from triplicate runs

**Fig. 6:**
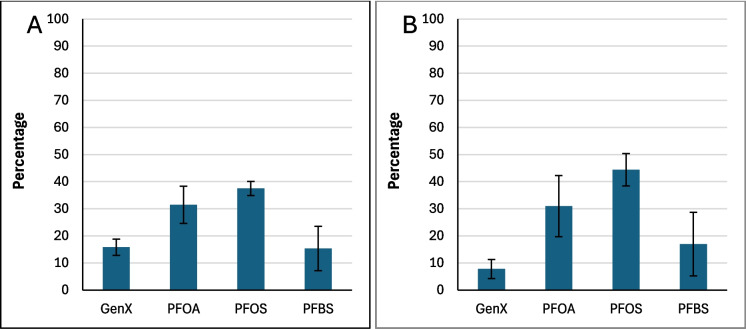
Panels A and B show the average percentage decomposition of the interactions of PFAS mixtures with the clay and the amendments of CMRIB, respectively, with respect to the different PFAS species. Mixtures comprised 6 GenX molecules, 6 PFOA molecules, 6 PFOS molecules, and 6 PFBS molecules. The average values are calculated from triplicate runs. Error bars denote standard deviation values calculated from triplicate runs

Figure 2 shows the average binding percentage probability of PFAS mixture calculated for each amended clay and the parent clay at pH 7. The amended clays showed slightly improved binding when compared to the parent clay, with the exception of CMCUR. CMCAF showed overall the highest binding to the PFAS mixture. CMCAF, CMCUR or CMRIB amendments could variably contribute to PFAS mixtures’ binding due to direct-assisted or indirect-assisted interactions (Fig. 2). Figure 3 presents the percentage contribution of each PFAS molecule binding to parent clay. Both PFOA and PFOS possessed approximate binding capacities (~ 40%). GenX and PFBS possessed the lowest binding capacities. Figure 4 presents the percentage contribution of each PFAS molecule bound through direct or direct-assisted interactions or indirectly to CMCAF (panels A, B, respectively). PFOA’s contribution to binding was the highest, for both direct and indirect binding. GenX contribution to binding was significantly increased at ~ 30% for both direct and indirect binding, when compared to the parent clay. Figure 5 presents the percentage contribution of each PFAS binding directly (through direct or direct-assisted interactions) or indirectly to CMCUR (panels A, B, respectively). PFOA and PFOS showed similar contributions to binding. Figure 6 presents the percentage contribution of each PFAS binding directly (through direct or direct-assisted interactions) or indirectly to CMRIB (panels A, B, respectively). PFOS showed the highest contribution to binding followed by PFOA, GenX and lastly PFBS.

### *Hydra vulgaris* Bioassay Result

**Fig. 7:**
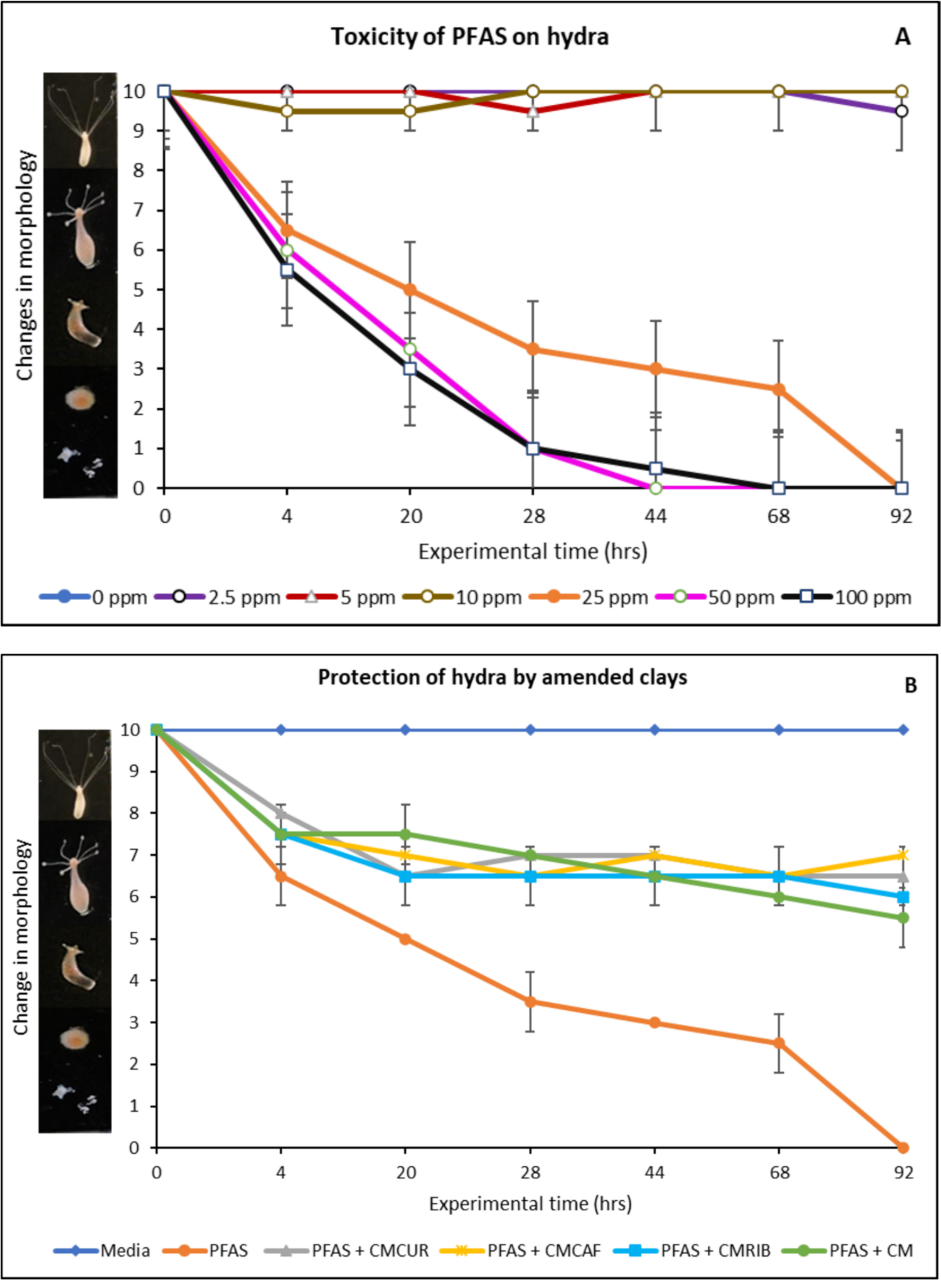
Toxicity of PFAS exposure to *Hydra vulgaris* (**A**) and protection from toxicity with amended clays (**B**)

The data in Fig. 7 showed the toxic effects of exposure to the PFAS mixture of PFOS, PFOA, PFBS, and GenX on *Hydra vulgaris* at concentrations ranging from 2.5 µg/mL to 100 µg/mL (Fig. 7A) and protection offered by the amended clays (Fig. 7B). At concentrations below 10 µg/mL, PFAS exposure to hydra did not induce a significant toxic effect on body morphology. It was observed that at higher concentrations (> 20 µg/mL), PFAS caused a dose-dependent toxicity to hydra. After 20 h of exposure, 25 µg/mL, 50 µg/mL and 100 µg/mL of PFAS exhibited a significant adverse effect of 50%, 65%, and 70% mortality when compared with the control. There was 100% mortality recorded in groups that were exposed to 50 µg/mL and 100 µg/mL of PFAS at 68 h and 44 h of exposure, respectively. However, treatment with different modified edible clays significantly mitigated the toxicities of PFAS. At 1% inclusion, CMCAF, CMCUR, and CMRIB offered 50% to 70% protection. CMCAF exhibited the highest protection of 70%, followed by CMCUR (65%) and CMRIB (60%). All the amended clays showed better protection than parent clay (CM) which offered 55% protection.

### *Lemna minor* Bioassay Result

**Fig. 8:**
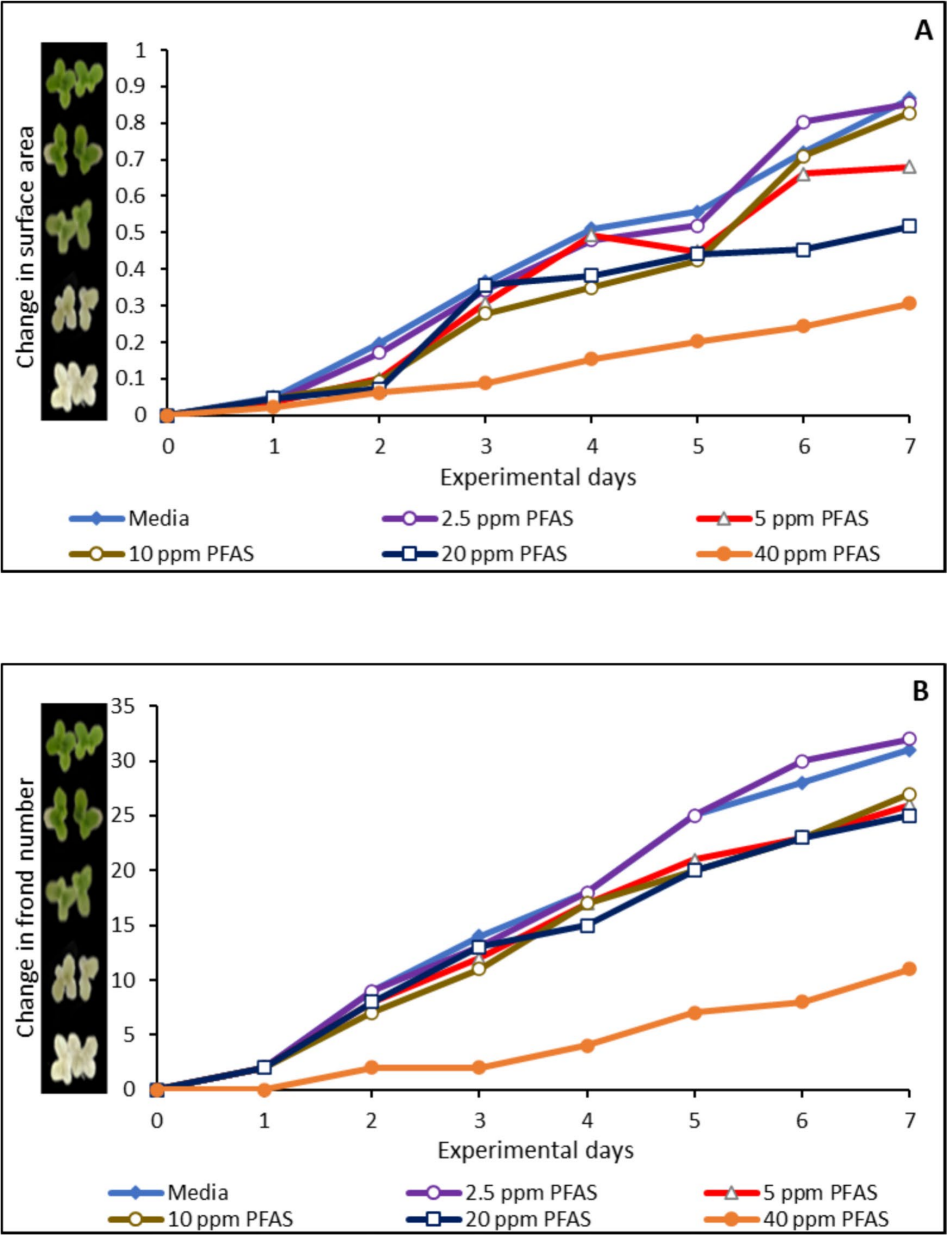

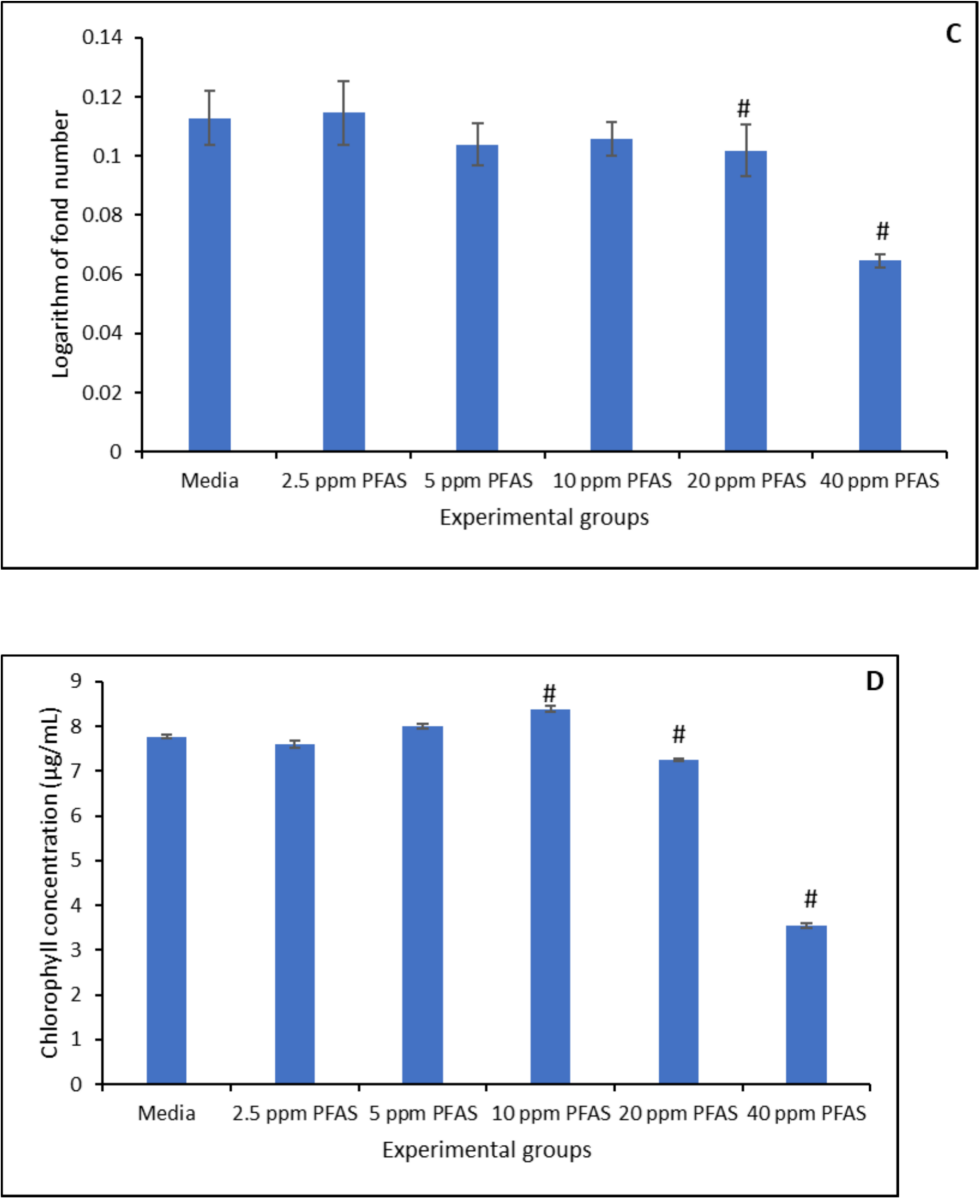
Toxicity of PFAS exposure to *Lemna minor* in terms of surface area (**A**), frond number (**B**), growth rate at day 7 (**C**), and chlorophyll content (**D**). ∗ Indicates a significant difference (p < 0.05) compared to the vehicle control group. Data is presented as mean ± standard deviation, n = 3

**Fig. 9:**
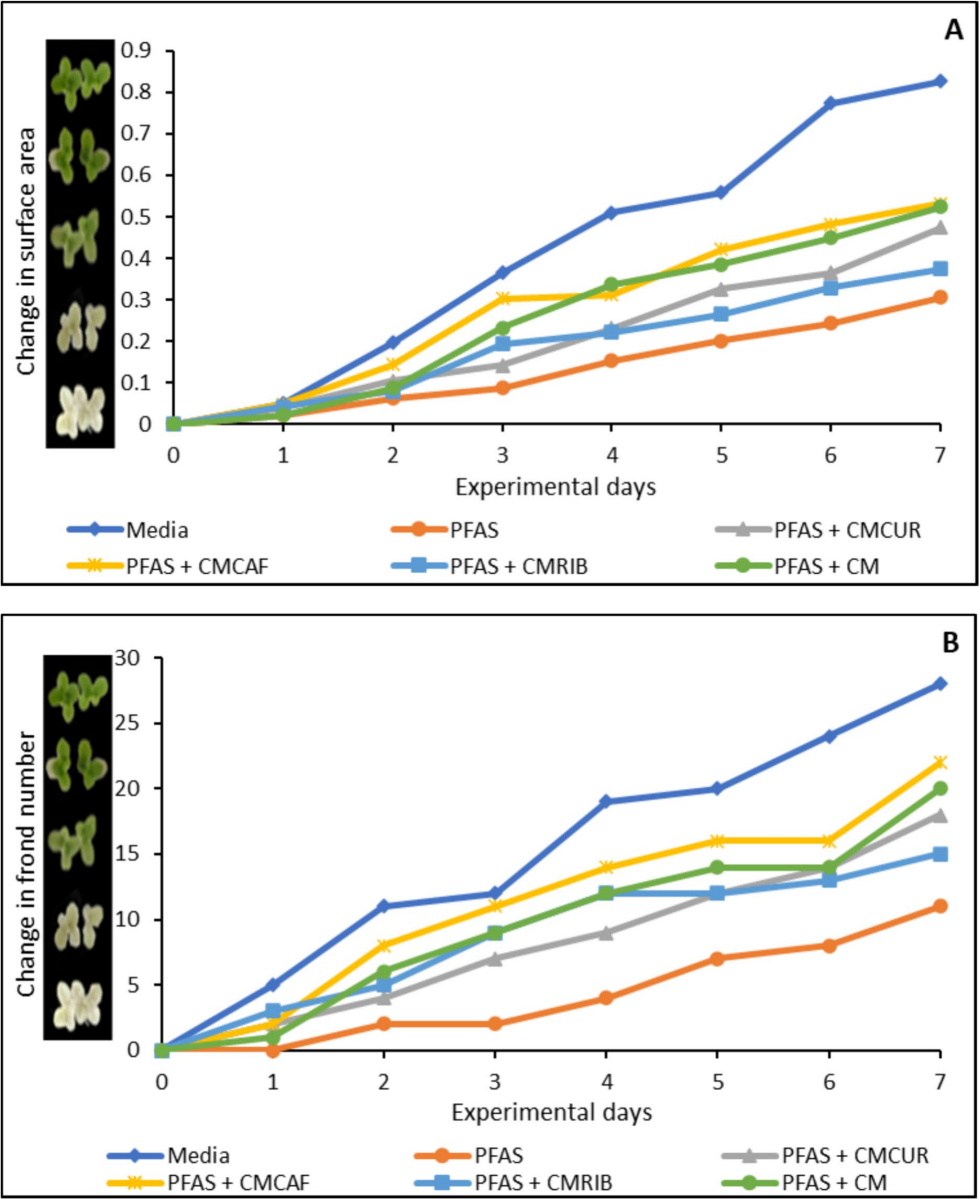

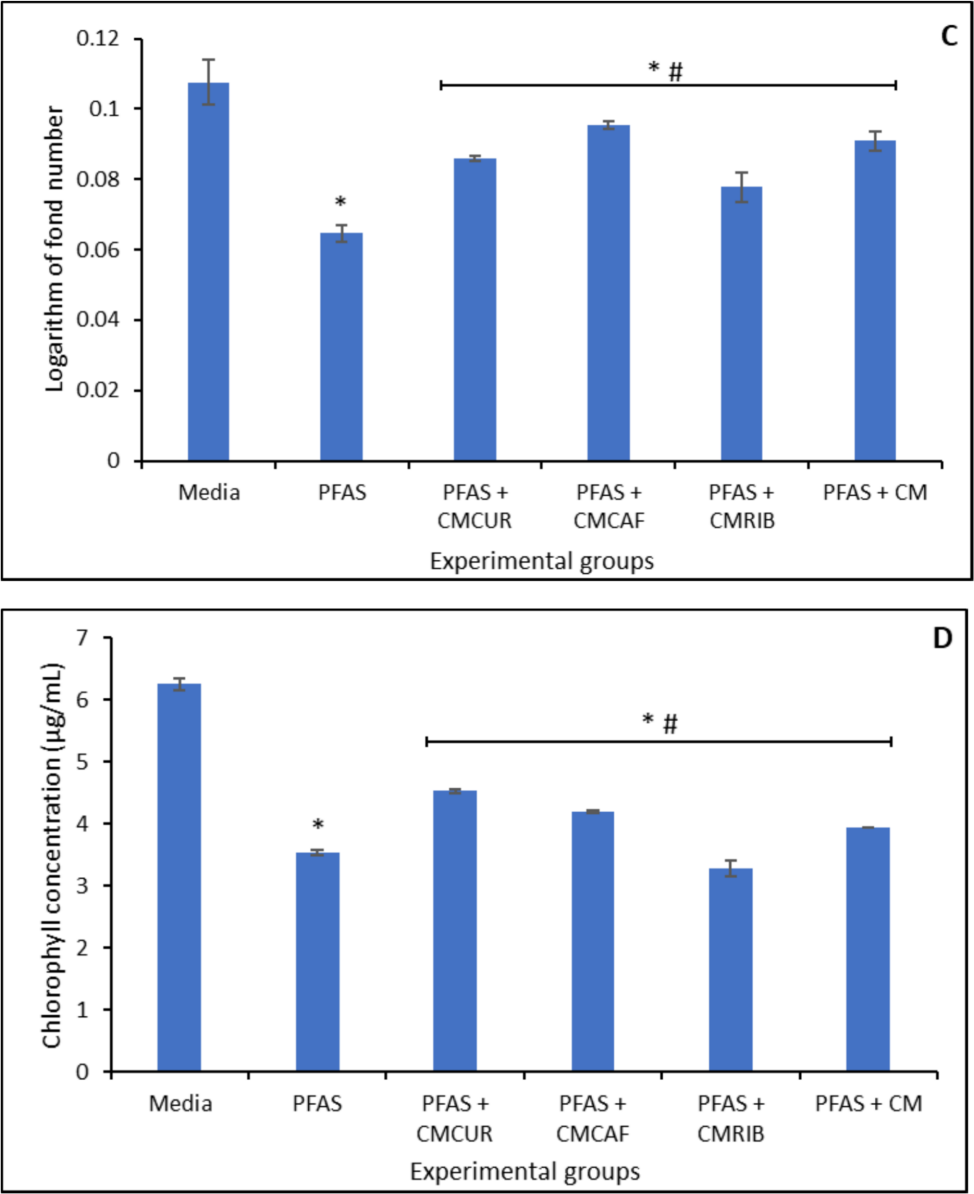
Protection of *Lemna minor* from PFAS toxicity with amended clays on surface area (**A**), frond number (**B**), growth rate at day 7 (**C**), and chlorophyll content (**D**). ∗ Indicates a significant difference (p < 0.05) compared to the vehicle control group. Data is presented as mean ± standard deviation, n = 3

The toxic effects of PFAS exposure on *Lemna minor* at concentrations ranging from 2.5 µg/mL to 40 µg/mL were shown in Fig. 8. At 40 µg/mL, PFAS exhibited a significant toxic effect to the growth of *Lemna minor* when compared with the control, causing 60%, 40%, 30%, 30%, and 40%, reduction on surface area (Fig. 8A), frond number (Fig. 8B), growth rate (logarithmic rate) (Fig. 8C), and chlorophyll concentration (Fig. 8D), respectively. At 20 µg/mL, PFAS caused toxic effects on *Lemna minor*, while at lower concentration PFAS did not demonstrate a significant toxic effect on the growth of *Lemna minor*. Treatment with the three modified clays used in this study significantly protected *Lemna minor* from growth stunting mediated by PFAS exposure. At 1% inclusion, CMCAF, CMCUR, and CMRIB enhanced the growth of *Lemna minor* by 16% to 30% protection. CMCAF demonstrated the highest protection of 47% (Fig. 9 A-D).

### *Caenorhabditis elegans* Bioassay Result

**Fig. 10:**
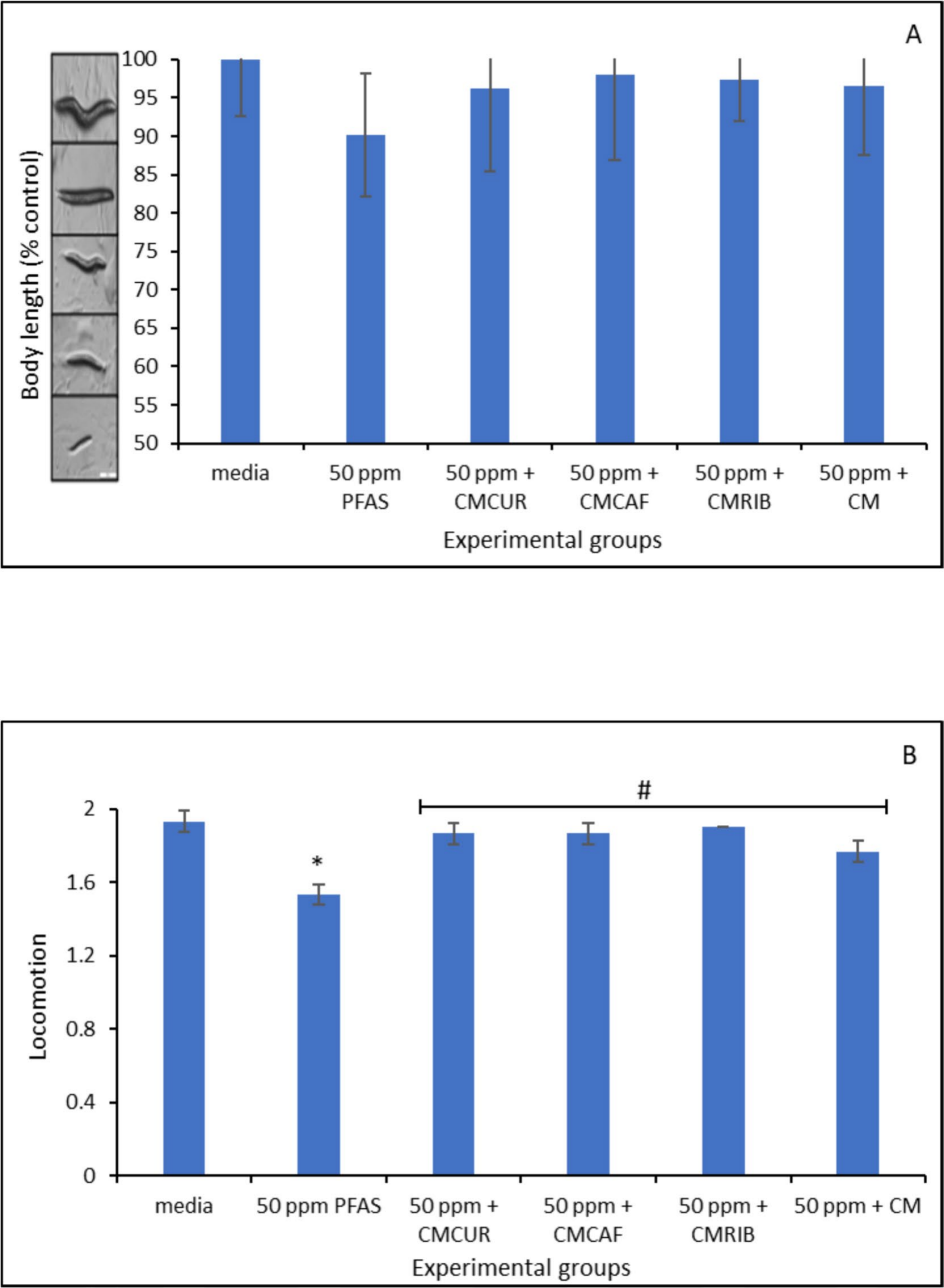

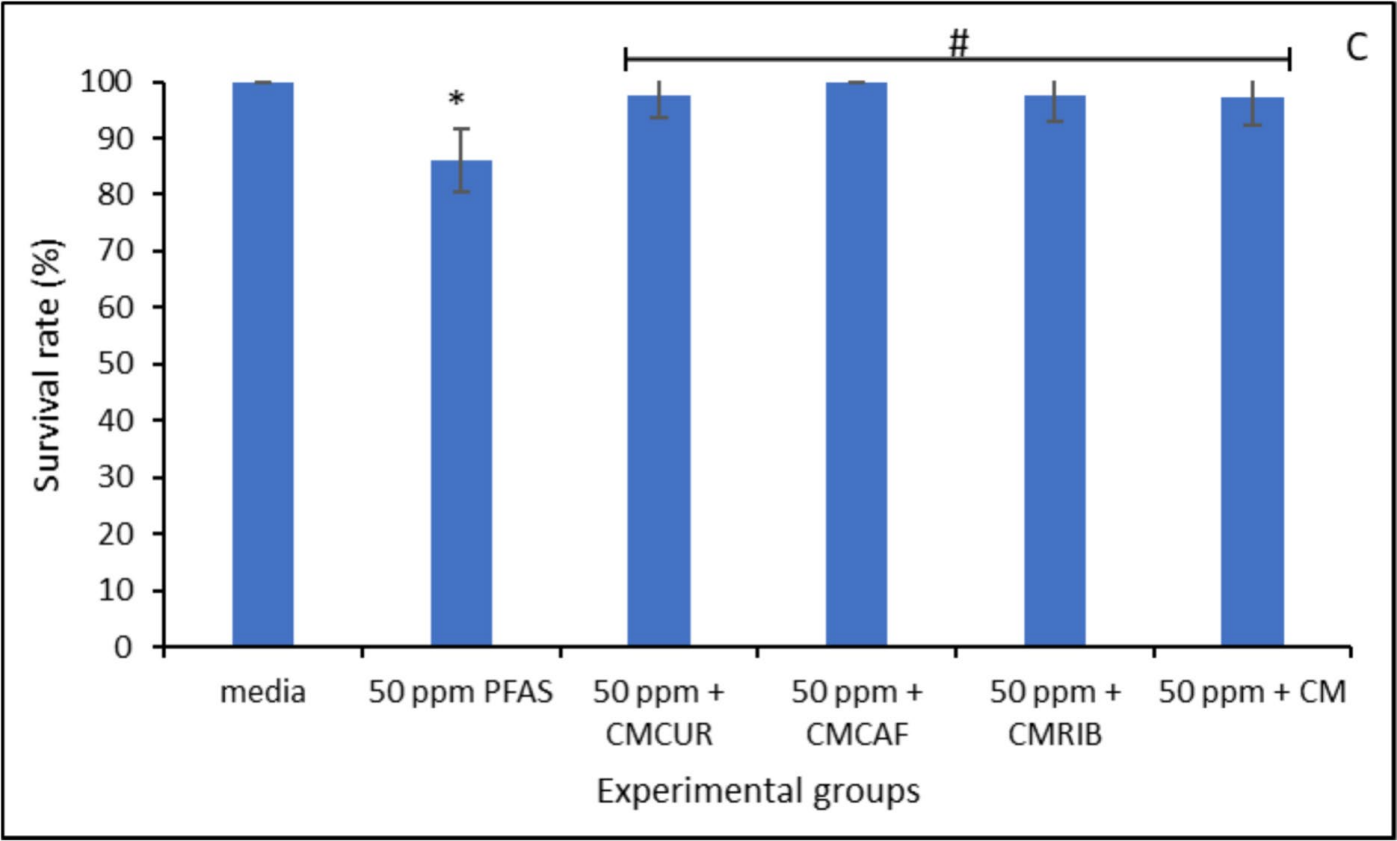
Toxicity of PFAS on body length (**A**), locomotion (**B**), and survival rate of Caenorhabditis elegans (**C**). Data represent the average value from triplicate analysis ± the standard deviation. * indicates a significant difference (p ≤ 0.05) compared to the vehicle control group

Figure 10 (A-C) showed the toxic effects of 50 µg/mL PFAS exposure on *Caenorhabditis elegans* and the mitigating effects of the amended clays. After *Caenorhabditis elegans* were exposed to 50 µg/mL of PFAS, there was a reduction in relative body length, survival rate and locomotion of nematodes compared to the control group. However, treatment with 1% (w/v) inclusion of the amended clay prevented these toxicities.

## Discussion

Throughout history, montmorillonite clays have been utilized for therapeutic purposes (Carrettero, 2002). They are currently gaining popularity as potential solutions to reduce exposure to harmful chemicals. Studies have shown that the adsorption efficacy of modified montmorillonite clays may vary depending on the amount and nature of added substances, which may also impact the spatial distribution of surface charges inside the montmorillonite interlayer space and potential interactions (Oladele et al., 2024; Wang et al., 2021). In this study montmorillonite clay was amended with three selected nutraceuticals to potentially improve adsorption performance of the clay for both short-chain and long-chain PFAS (based on computational predictions). The results of this study revealed that the amended clays were good sorbents for PFOA, PFOS, PFBS and GenX. The findings of this study also demonstrated that the common nutraceuticals, curcumin, riboflavin, and caffeine, increased the activity of parent montmorillonite for PFAS moieties and their functional groups. The results of this investigation are consistent with other published research that has demonstrated the enhanced binding ability of clay after various amendments (Dong et al., 2024; Jiang et al., 2023; Zhou et al., 2024). Furthermore, previous research from our lab have found that clays have a higher capacity to bind PFAS after being amended with nutrients like choline and carnitine (Wang et al., 2021).

Adsorption isotherms are important to comprehend the interaction between PFAS molecules and the active surfaces of amended clays. These isotherms can be used to determine equilibrium conditions, capacities, and efficiencies of the adsorption process. The Langmuir isotherm was the best adsorption model for PFOS, PFOA, and GenX suggesting that they use a monolayer interaction mechanism to bind to the surface of parent and amended clays during the adsorption process. However, the Freundlich isotherm, which assumes multilayer adsorption, was the best adsorption model for PFBS. The (1/n) values, which provide details on the adsorption intensity and surface heterogeneity, can be utilized as a predictor of the viability and favorability of adsorption (Mukherjee et al., 2019). The value of 1/n denotes three different isotherm types: unfavorable (1/n > 1), favorable (0 < 1/n < 1), and irreversible (1/n = 0) (Chernomorova et al., 2023). A good adsorption process was indicated by the (1/n) values of PFBS in this experiment, which were between 0 and 1.

Additionally, the reactions between each of the four PFAS compounds and the clays had significant Gibbs free energies for their binding interactions. This indicated that their interactions were thermodynamically favorable. These results supported the in silico studies. Within the simulations of PFAS with parent clay, longer-chained PFAS (PFOA and PFOS) possessed higher binding probabilities compared to GenX and PFBS, suggesting that the length of the perfluoroalkyl tail and the hydrophobic interactions of the tail play a critical role in binding. All four PFAS species interacted with the clay through their perfluoroalkyl tail in nearly all of the binding instances. The GenX functional group (–COO^−^) was facing outwards to the solvent at ~ 93% of the binding instances. The PFOA functional group (–COO^−^) was facing outwards to the solvent at ~ 96% of the binding instances. The PFOS functional group (–SO^−^_3_) was facing outwards to the solvent at ~ 82% of the binding instances. The PFBS functional group (–SO^−^_3_) was facing outwards to the solvent at ~ 83% of the binding instances. Clusters of PFAS molecules binding to clay were very rare (~ 0.3%), however aggregation was sometimes observed in the water box mainly between PFOS-PFOS, followed by PFOA-PFOA, or PFOS-PFOA. GenX was participating in aggregation to a lesser degree with other PFAS species, while PFBS was very rarely found interacting with other PFAS species.

**Fig. 11:**
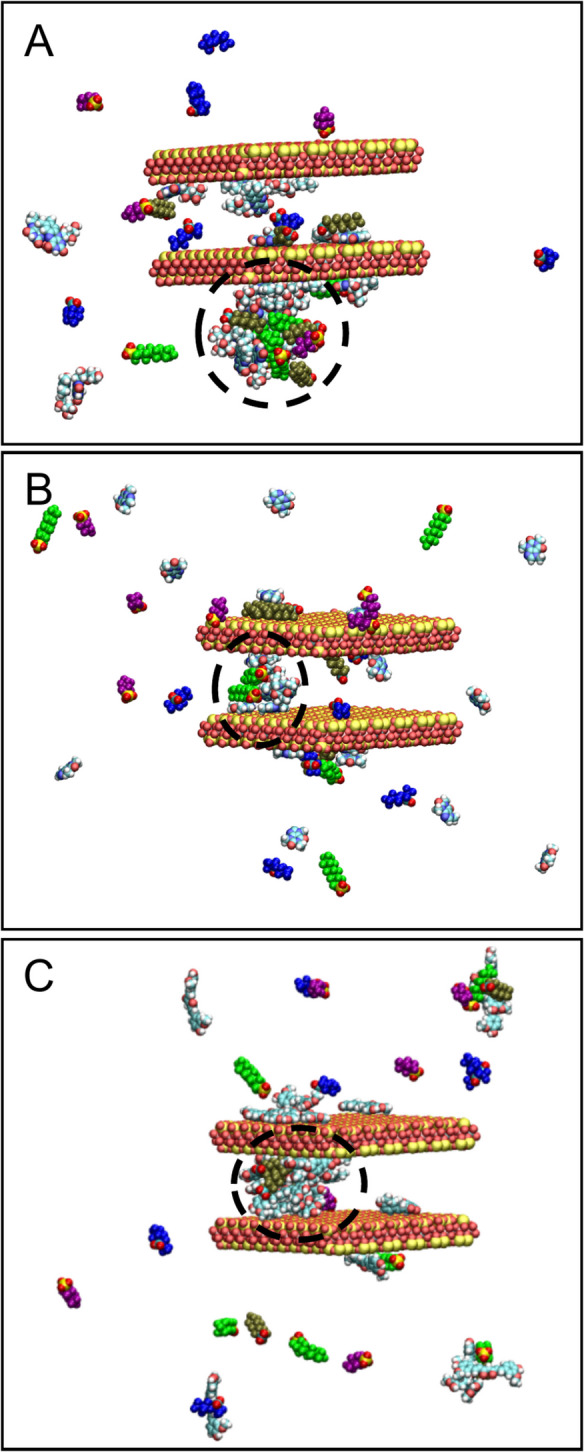
Panels **A**–**C** show the PFAS mixture in complex with the CMRIB, CMCAF, and CMCUR, respectively. The clay layers and caffeine amendments are shown in vdW representation, coloured by atom type. The PFAS are shown in vdW representation and their perfluoroalkyl tails are coloured in blue, tan, green, and purple for GenX, PFOA, PFOS and PFBS, respectively. The functional groups of PFAS are coloured by atom type. Hydrogen atoms of parent clay were omitted for clarity. The encircled molecules with dashed line are discussed in the text

Importantly, caffeine-, riboflavin-, and curcumin-amended clay protected *Lemna minor* against PFAS-induced toxicities and growth stunting. This finding supports an earlier report that riboflavin modulates plant somatic embryogenesis and promotes plant development (Xu et al., 2023a, b). Riboflavin-amended clay may have served as a non-enzymatic antioxidant to lessen the harmful effects of PFAS on Lemna minor because oxidative stress has been linked to PFAS toxicity. According to previous studies, defense mechanism against ROS is made up of both enzymatic and non-enzymatic components that reduce oxidative stress and support the growth and development of plants (Das & Roychoudhury, 2014). Riboflavin can reduce biotic and abiotic stressors (Deng et al., 2013, Rhaman et al., 2021). Flavin mononucleotide and flavin adenine dinucleotide, which are significant flavoproteins and flavoenzymes respectively, are precursors of riboflavin and are crucial for cellular metabolism (Fischer & Bacher, 2008; Haase et al., 2014). Flavin-dependent proteins which belong to the family of flavoenzymes and flavoproteins, work in redox capacity by absorbing electrons from a donor substrate and moving them to an acceptor matrix (Ferreira et al., 2014). Flavoenzymes aid in the manufacture of abscisic acid in plants (Seo et al., 2000) and promote plant growth and development through the modulation of flavonoids and hormones (Barrero et al., 2005). Cell signaling, coenzyme function, and antioxidant activity are all aspects of riboflavin metabolism (Pinto & Zempleni, 2016). Within the simulation studies, the PFAS mixture in complex with CMRIB behaved similarly to the parent clay simulations, with respect to the fact that PFOS showed the highest contribution to binding followed by PFOA, GenX and lastly PFBS. GenX interacted primarily via its perfluoroalkyl tail with riboflavin (~ 90%). However, PFOA, PFOS and PFBS showed a significant tendency to interact with riboflavin with both their perfluoroalkyl tail and functional groups at pH 7 (~ 30%, ~ 30%, and ~ 45%, respectively). Riboflavin’s polar groups were mainly participating in the interactions with all PFAS species (~ 95%). Example cases corresponding to selected snapshots of a particular simulation of PFAS mixture and CMRIB are presented in Fig. 11. The encircled molecules in Panel A correspond to riboflavin clumps that attract PFAS molecules with which they interact primarily with hydrogen bonds.

Interestingly, CMCAF offered the highest protection for *Hydra vulgaris* (in neutral media at pH 7) and *Lemna minor* (in slight acidic media at pH 5.4). This corroborates the outcomes of the simulation studies. For CMCAF, PFOA’s contribution to binding was the highest, for both direct binding and indirect binding, as shown in Fig. 4. Additionally, GenX contribution to binding was significantly increased at ~ 30% for both direct and indirect binding. GenX interacted primarily via its perfluoroalkyl tail only (~ 93%) with caffeine’s non-polar (~ 99%) and polar groups (~ 25%). Similarly, PFOA interacted primarily via its perfluoroalkyl tail only (~ 80%) or both its perfluoroalkyl tail and functional group (~ 20%) with caffeine’s non-polar (~ 99%) and polar groups (~ 35%). PFOS interacted via its perfluoroalkyl tail only (~ 75%) or both its perfluoroalkyl tail functional groups (~ 25%) with caffeine’s non-polar (~ 99%) and polar groups (~ 45%). PFBS was rarely found to interact with CMCAF, despite the fact that its binding mechanisms were similar to PFOS; this suggests the important role of hydrophobicity in conjunction with the perfluoroalkyl tail length. The change in the binding contributions of PFOA and PFOS within the mixture, in comparison to parent clay and other amended clays, may be attributed to the fact that PFOA and PFOS are very similar molecules and potentially bind antagonistically to caffeine amendments. According to visual inspection, PFOS shows a slightly higher tendency to aggregate with other molecules compared to PFOA. Additionally, according to our analysis PFOS shows a higher tendency to interact both with its perfluoroalkyl tail and functional group, which might not be favorable in the case of caffeine. Due to caffeine’s smaller size there are less “interaction sites” compared to the other two amended clays, which may be more favored to be occupied by PFOA. Example cases corresponding to selected snapshots of a particular simulation of PFAS mixture and CMCAF are presented in Fig. 11. The encircled molecules in Panels B correspond to a cluster of PFAS molecules which interact with caffeine amendments primarily hydrophobically.

PFAS have been detected in aquatic environments including surface waters (rivers, lakes and oceans), groundwater and marine ecosystems. In this study, the *Hydra vulgaris* model was used to evaluate both the toxicity of PFAS and possible protective effects of the amended clays. *Hydra vulgaris* is a simple aquatic organism which serves as a valuable model in toxicological studies due to its sensitivity to various environmental chemical exposures. The results of this study demonstrated that combined exposure of PFOS, PFOA, PFBS, and GenX at concentration at 25 µg/mL had a significant toxicity on the body morphology of *Hydra vulgaris*. This agrees with previous study which showed that both single and combined exposure of PFOA and PFOS had marked toxicity on Hydra (Wang et al., 2021). However, treatment with riboflavin-, caffeine-, and curcumin-amended clays significantly reduced the toxicities associated with PFAS exposure. This showed that the amended clays can significantly adsorb the four PFAS and reduce their exposure and associated toxicities. Curcumin exhibits significant biological activities including antioxidative, anti-inflammatory, and modulatory properties, however, specific studies examining effects of curcumin on hydra vulgaris are limited. Since oxidative stress and inflammatory activity have been implicated as the underlying mechanism for PFAS toxicity, curcumin amended clay may have protected hydra vulgaris against PFAS toxicity via its renowned antioxidant and anti-inflammatory activities.

Within the simulation studies, the PFAS mixture in complex with CMCUR behaved similarly to the parent clay simulations, with respect to the fact that PFOS showed the highest contribution to binding followed by PFOA, GenX and lastly PFBS. GenX interacted primarily via its perfluoroalkyl tail (~ 85%) with both curcumin’s non-polar (~ 70%) and polar groups (~ 80%). Similarly, PFOA interacted primarily via its perfluoroalkyl tail only (~ 90%) with both curcumin’s non-polar (~ 60%) and polar groups (~ 85%). PFOS interacted with its perfluoroalkyl tail only (~ 70%) or both its perfluoroalkyl tail and functional group (~ 30%) with both curcumin’s non-polar (~ 70%) and polar groups (~ 90%). PFBS was rarely found to interact with CMCUR, however its binding mechanisms were similar to PFOS. Due to curcumin’s tendency to create big clumps the likelihood of the PFAS mixtures interacting with all curcumin’s chemical groups was increased. Furthermore, according to visual inspection, curcumin molecules were stacking on top of each other having their polar edges exposed and more “available” for interactions with PFAS. Example cases corresponding to selected snapshots of a particular simulation of the PFAS mixture and CMCUR are presented in Fig. 11. The encircled molecules in Panel C correspond to curcumin clumps that attract PFAS molecules with which they interact both with hydrophobic interactions and hydrogen bonds.

Exposure of PFAS to *Caenorhabditis elegans* caused various toxicities including shortened body length, altered locomotion, and responses. However, the amended clays significantly protected the treated *Caenorhabditis elegans* from PFAS mediated toxicities. This indicated that the amended clays effectively adsorbed the four PFAS thus reducing their exposure to the worm. A previous experimental study has shown that riboflavin enhances the activity of complexes I and IV in *C. elegans*, resulting in decreased lactic acidosis and oxidative stress as well as improved metabolic function (Grad & Lemire, 2006). Similarly, in an aged *Caenorhabditis elegans* model, long-term caffeine consumption has been found to have protective effects on intestinal ageing and mitochondrial function. Research has shown that curcumin is effective in reducing heat stress-induced damage from free radicals by strengthening the antioxidant system in *C. elegans* (Xu et al., 2023a, b). Furthermore, supplements containing curcumin can increase the lifespan of Caenorhabditis elegans. According to Liao et al. (2011), this impact is ascribed to its antioxidant qualities, which lessen the buildup of lipofuscin and intracellular reactive oxygen species (ROS).

## Conclusion

PFAS have been shown to accumulate in the human body. Thus, development of enterosorbents that will prevent systemic absorption and bioaccumulation of PFAS could be a viable strategy to mitigate exposures via contaminated water and food. This study investigated the adsorption and detoxification capacity of caffeine-, riboflavin-, and curcumin-amended montmorillonite clay. Using adsorption isotherms and computational modeling, the capacity of the nutraceutical-amended clays to absorb PFAS was evaluated. Both the in vitro and in silico molecular dynamics simulations demonstrated that amendment of the clay enhanced the binding capacity for the four PFAS (PFOS, PFOA, PFBS and GenX). Furthermore, the simulations elucidated the binding contribution of each PFAS molecule to parent and amended clays as well as uncovered how amended clays can contribute to enhanced binding of different PFAS in the mixture. According to computational modelling, hydrogen bonds and electrostatic interactions were the main adsorption processes. In vivo data established the proof of concept and protective ability of the amended clays against PFAS exposure.

Caffeine, riboflavin and curcumin are classified as nutraceuticals. Our results showed that nutraceutical-amended montmorillonite clays have a considerable capacity to bind and detoxify PFAS. This suggests that consuming edible clay-based sorbents could dramatically reduce the amount of PFAS toxicity that humans and animals are exposed to through contaminated food and water. The protection offered by amended clays could be attributed to both their enhanced PFAS binding capacity and biological activities of these nutraceuticals (caffeine, riboflavin, and curcumin) including antioxidative, anti-inflammatory and modulatory activities which mitigate the oxidative stress and inflammatory effects of PFAS. Based on our earlier findings in animals, long-term consumption of clay levels up to 2% of the diet did not result in any negative effects on blood biochemistry, body weights, or histology (Afriyie-Gyawu et al., 2005; Marroquin-Cardona et al., 2011). These findings imply that there is no observable buildup, or detectable toxicity of montmorillonite at doses that are effective against PFAS. Importantly, nutraceutical-amended clays can eventually be included in the diet of humans and animals or added in flavored water, tablets, capsules, a variety of foods, snacks, and condiments to decrease toxin bioavailability and exposures to PFAS (PFOS, PFOA, PFBS and GenX). More studies are required to confirm the efficacy and safety of these clays in higher animals.

## Supplementary Information

Below is the link to the electronic supplementary material.

Supplementary file1 (PDF 4798 KB)

## Data Availability

Data will be available on request.

## References

[CR1] Afriyie-Gyawu, E., Mackie, J., Dash, B., Wiles, M., Taylor, J., Huebner, H., Tang, L., Guan, H., Wang, J. S., & Phillips, T. (2005). Chronic toxicological evaluation of dietary NovaSil clay in Sprague-Dawley rats. *Food Additives and Contaminants.,**22*(3), 259–69. 16019794 10.1080/02652030500110758

[CR2] Ahrens, L., & Bundschuh, M. (2014). Fate and effects of poly- and perfluoroalkyl substances in the aquatic environment: A review. *Environmental Toxicology and Chemistry,**33*(9), 1921–1929. 10.1002/etc.2582 24924660 10.1002/etc.2663

[CR3] Barrero, J. M., Piqueras, P., González-Guzmán, M., Serrano, R., Rodríguez, P. L., Ponce, M. R., & Micol, J. L. (2005). A mutational analysis of the ABA1 gene of *Arabidopsis thaliana* highlights the involvement of ABA in vegetative development. *Journal of Experimental Botany,**56*(417), 2071–2083. 10.1093/jxb/eri206 15983017 10.1093/jxb/eri206

[CR4] Boyer, T. H., Fang, Y., Ellis, A., Dietz, R., Choi, Y. J., Schaefer, C. E., Higgins, C. P., & Strathmann, T. J. (2021). Anion exchange resin removal of per- and polyfluoroalkyl substances (PFAS) from impacted water: A critical review. *Water Research,**200*, 117244. 10.1016/j.watres.2021.117244 34089925 10.1016/j.watres.2021.117244

[CR5] Brase, R. A., Mullin, E. J., & Spink, D. C. (2021). Legacy and emerging per- and polyfluoroalkyl substances: Analytical techniques, environmental fate, and health effects. *International Journal of Molecular Sciences,**22*(2), 995. 10.3390/ijms22020995 33498193 10.3390/ijms22030995 PMC7863963

[CR6] Brooks, B. R., Brooks, C. L., III, Mackerell, A. D., Jr, Nilsson, L., Petrella, R. J., Roux, B., Won, Y., Archontis, G., Bartels, C., Boresch, S., Caflisch, A., Caves, L., Cui, Q., Dinner, A. R., Feig, M., Fischer, S., Gao, J., Hodoscek, M., Im, W., Kuczera, K., … Karplus, M. (2009). CHARMM: The biomolecular simulation program. *Journal of Computational Chemistry, 30*(10), 1545–1614. 10.1002/jcc.21287 10.1002/jcc.21287 PMC2810661 19444816

[CR7] Buck, R. C., et al. (2011). Perfluoroalkyl and polyfluoroalkyl substances in the environment: Terminology, classification, and origins. *Integrated Environmental Assessment and Management,**7*(4), 513–541. 10.1002/ieam.258 21793199 10.1002/ieam.258 PMC3214619

[CR8] Carretero, M. I. (2002). Clay minerals and their beneficial effects upon human health: A review. *Applied Clay Science,**21*, 155–163. 10.1016/S0169-1317(01)00085-0

[CR9] Chernomorova, M. A., Myakinina, M. S., Zhinzhilo, V. A., & Uflyand, I. E. (2023). Analytical determination of cephalosporin antibiotics using coordination polymer based on cobalt terephthalate as a sorbent. *Polymers,**15*, 548. 10.3390/polym15020548 36771849 10.3390/polym15030548 PMC9919266

[CR10] Cousins, I. T., et al. (2020). The high persistence of PFAS is sufficient for their management as a chemical class. *Environmental Science: Processes & Impacts,**22*(12), 2307–2312. 10.1039/d0em00291j 33230514 10.1039/d0em00355g PMC7784706

[CR11] Das, K., & Roychoudhury, A. (2014). Reactive oxygen species (ROS) and response of antioxidants as ROS-scavengers during environmental stress in plants. *Frontiers in Environmental Science,**2*, 53. 10.3389/fenvs.2014.00053

[CR12] Deng, B., Jin, X., Yang, Y., Lin, Z., & Zhang, Y. (2013). The regulatory role of riboflavin in the drought tolerance of tobacco plants depends on ROS production. *Plant Growth Regulation,**72*, 269–277. 10.1007/s10725-013-9858-8

[CR13] Dimitrakopoulou, M. E., Karvounis, M., & Marinos, G. (2024). Comprehensive analysis of PFAS presence from environment to plate. *npj Sci Food,**8*, 80. 10.1038/s41538-024-00319-1 39369000 10.1038/s41538-024-00319-1 PMC11455986

[CR14] Dixit, F., Dutta, R., Barbeau, B., Berube, P., & Mohseni, M. (2021). PFAS removal by ion exchange resins: A review. *Chemosphere,**272*, 129777. 10.1016/j.chemosphere.2021.129777 33582507 10.1016/j.chemosphere.2021.129777

[CR15] Dong, Q., Min, X., Zhao, Y., & Wang, Y. (2024). Adsorption of per- and polyfluoroalkyl substances (PFAS) by ionic liquid-modified clays: Effect of clay composition and PFAS structure. *Journal of Colloid and Interface Science,**654*, 925–934. 10.1016/j.jcis.2023.10.112 37898076 10.1016/j.jcis.2023.10.112

[CR16] Drost, W., Matzke, M., & Backhaus, T. (2007). Heavy metal toxicity to lemna minor: Studies on the time dependence of growth inhibition and the recovery after exposure. *Chemosphere,**67*, 36–43. 17157350 10.1016/j.chemosphere.2006.10.018

[CR17] Eastman, P., Swails, J., Chodera, J. D., McGibbon, R. T., Zhao, Y., Beauchamp, K. A., Wang, L. P., Simmonett, A. C., Harrigan, M. P., Stern, C. D., Wiewiora, R. P., Brooks, B. R., & Pande, V. S. (2017). OpenMM 7: Rapid development of high performance algorithms for molecular dynamics. *PLOS Computational Biology,**13*(7), e1005659. 10.1371/journal.pcbi.1005659 28746339 10.1371/journal.pcbi.1005659 PMC5549999

[CR18] Fenton, S. E., et al. (2021). Per- and polyfluoroalkyl substance toxicity and human health review: Current state of knowledge and strategies for informing future research. *Environmental Toxicology and Chemistry,**40*(3), 606–630. 10.1002/etc.5067 33017053 10.1002/etc.4890 PMC7906952

[CR19] Ferreira, P., Martínez-Júlvez, M., & Medina, M. (2014). Electron transferases. In *Flavins and flavoproteins: Methods and protocols* (pp. 79–94). Springer. 10.1007/978-1-4939-0787-7_4 10.1007/978-1-4939-0452-5_5 24764089

[CR20] Fischer, M., & Bacher, A. (2008). Biosynthesis of vitamin B2: Structure and mechanism of riboflavin synthase. *Archives of Biochemistry and Biophysics,**474*(2), 252–265. 10.1016/j.abb.2008.02.008 18298940 10.1016/j.abb.2008.02.008

[CR21] Gebbink, W. A., & van Leeuwen, S. P. J. (2020). Environmental contamination and human exposure to PFASs near a fluorochemical production plant: Review of historic and current PFOA and GenX contamination in the Netherlands. *Environmental International,**137*, 105583. 10.1016/j.envint.2020.105583 10.1016/j.envint.2020.105583 32106048

[CR22] Grad, L. I., & Lemire, B. D. (2006). Riboflavin enhances the assembly of mitochondrial cytochrome c oxidase in *C*. *elegans* NADH-ubiquinone oxidoreductase mutants. *Biochimica et Biophysica Acta (BBA) – Bioenergetics,**1757*(2), 115–122. 10.1016/j.bbabio.2005.11.009 16443191 10.1016/j.bbabio.2005.11.009

[CR23] Grant, P. G., & Phillips, T. D. (1998). Isothermal adsorption of aflatoxin B (1) on HSCAS clay. *Journal of Agricultural and Food Chemistry,**46*, 599–605. 10554284 10.1021/jf970604v

[CR24] Haase, I., Gräwert, T., Illarionov, B., Bacher, A., & Fischer, M. (2014). Recent advances in riboflavin biosynthesis. In *Flavins and flavoproteins: Methods and protocols* (pp. 15–40). Springer. 10.1007/978-1-4939-0787-7_2 10.1007/978-1-4939-0452-5_2 24764086

[CR25] He, Y., et al. (2022). Human exposure to F-53B in China and the evaluation of its potential toxicity: An overview. *Environmental International,**161*, 107108. 10.1016/j.envint.2022.107108 10.1016/j.envint.2022.107108 35121495

[CR26] Heinz, H., Lin, T. J., Mishra, R. K., & Emami, F. S. (2013). Thermodynamically consistent force fields for the assembly of inorganic, organic, and biological nanostructures: The INTERFACE force field. *Langmuir,**29*, 1754–1765. 23276161 10.1021/la3038846

[CR27] IARC Working Group on the Evaluation of Carcinogenic Risks to Humans. (2017). *IARC Monographs on the Evaluation of Carcinogenic Risks to Humans, No. 110*. Retrieved September 23 2024, from https://monographs.iarc.who.int/list-of-classifications/

[CR28] Interstate Technology & Regulatory Council (ITRC). (2022). *PFAS fact sheets*. ITRC.

[CR29] Jiang, T., Zhang, W., Ilang, A. K., Feldblyum, J. I., Wei, Z., et al. (2023). Surfactant-modified clay for adsorption of mixtures of per- and polyfluoroalkyl substances (PFAS) in aqueous solutions. *ACS Applied Engineering Materials,**1*, 394–407. 10.1021/acsaem.3c01188

[CR30] Jo, S., Kim, T., Iyer, V. G., & Im, W. (2008). CHARMM-GUI: A web-based graphical user interface for CHARMM. *Journal of Computational Chemistry,**29*(11), 1859–1865. 10.1002/jcc.20945 18351591 10.1002/jcc.20945

[CR31] Joo, S. H., Liang, Y., Kim, M., Byun, J., & Choi, H. (2021). Microplastics with adsorbed contaminants: Mechanisms and treatment. *Environmental Challenges,**3*, 100042. 10.1016/j.envc.2021.100042 37521158 10.1016/j.envc.2021.100042 PMC9767417

[CR32] Kissa, E. (2001). *Fluorinated surfactants and repellents* (2nd ed., rev. and expanded). Marcel Dekker.

[CR33] Kumar, A., Hodnett, B. K., Hudson, S., & Davern, P. (2020). Modification of the zeta potential of montmorillonite to achieve high active pharmaceutical ingredient nanoparticle loading and stabilization with optimum dissolution properties. *Colloids and Surfaces B: Biointerfaces,**193*, 111120. 10.1016/j.colsurfb.2020.111120 32505995 10.1016/j.colsurfb.2020.111120

[CR34] Lee, J., Cheng, X., Swails, J. M., Yeom, M. S., Eastman, P. K., Lemkul, J. A., Wei, S., Buckner, J., Jeong, J. C., Qi, Y., Jo, S., Pande, V. S., Case, D. A., Brooks, C. L., III., Mackerell, A. D., Jr., Klauda, J. B., & Im, W. (2016). CHARMM-GUI input generator for NAMD, GROMACS, AMBER, OpenMM, and CHARMM/OpenMM simulations using the CHARMM36 additive force field. *Journal of Chemical Theory and Computation,**12*(1), 405–413. 10.1021/acs.jctc.5b00935 26631602 10.1021/acs.jctc.5b00935 PMC4712441

[CR35] Lewis, R. C., Johns, L. E., & Meeker, J. D. (2015). Serum biomarkers of exposure to perfluoroalkyl substances in relation to serum testosterone and measures of thyroid function among adults and adolescents from NHANES 2011–2012. *International Journal of Environmental Research and Public Health,**12*(6), 6098–6114. 10.3390/ijerph120606098 26035660 10.3390/ijerph120606098 PMC4483690

[CR36] Li, Z., Luo, Z. M., Huang, Y., Wang, J. W., & Ouyang, G. (2023). Recent trends in degradation strategies of PFOA/PFOS substitutes. *Chemosphere,**315*, 137653. 10.1016/j.chemosphere.2023.137653 36581124 10.1016/j.chemosphere.2022.137653

[CR37] Liao, V. H., Yu, C. W., Chu, Y. J., Li, W. H., Hsieh, Y. C., & Wang, T. T. (2011). Curcumin-mediated lifespan extension in *Caenorhabditis elegans*. *Mechanisms of Ageing and Development,**132*(10), 480–487. 10.1016/j.mad.2011.07.008 21855561 10.1016/j.mad.2011.07.008

[CR38] Lohmann, R., et al. (2020). Are fluoropolymers really of low concern for human and environmental health and separate from other PFAS? *Environmental Science & Technology,**54*(19), 12820–12828. 10.1021/acs.est.0c02910 33043667 10.1021/acs.est.0c03244 PMC7700770

[CR39] Marroquin-Cardona, A., Deng, Y., Garcia-Mazcorro, J., Johnson, N. M., Mitchell, N., Tang, L., Robinson, A., Taylor, J., Wang, J. S., & Phillips, T. D. (2011). Characterization and safety of uniformparticle size novasil clay as a potential aflatoxin enterosorbent. *Applied Clay Science,**54*(3–4), 248–257. 22249378 10.1016/j.clay.2011.09.009 PMC3253772

[CR40] Mukherjee, M., Goswami, S., Banerjee, P., Sengupta, S., Das, P., Banerjee, P. K., & Datta, S. (2019). Ultrasonic assisted graphene oxide nanosheet for the removal of phenol containing solution. *Environmental Technology & Innovation,**13*, 398–407.

[CR41] Oladele, J. O., Wang, M., Xenophontos, X., Kendall, L., Tamamis, P., & Phillips, T. D. (2024). Chlorophyll-amended organoclays for the detoxification of ochratoxin A. *Toxins,**16*(11), 1–32. 10.3390/toxins16110479 10.3390/toxins16110479 PMC11598794 39591234

[CR42] Organisation for Economic Co-operation and Development (OECD). (2018). *Toward a new comprehensive global database of per- and polyfluoroalkyl substances (PFASs): Summary report on updating the OECD 2007 list of per- and polyfluoroalkyl substances* (PFASs). OECD.

[CR43] Pinto, J. T., & Zempleni, J. (2016). Riboflavin. *Advances in Nutrition,**7*(6), 973–975. 10.3945/an.116.012716 27633112 10.3945/an.116.012716 PMC5015041

[CR44] Rahman, M. S., Imran, S., Karim, M. M., Chakrobortty, J., Mahamud, M. A., Sarker, P., Tahjib-Ul-Arif, M., Robin, A. H. K., Ye, W., Murata, Y., & Hasanuzzaman, M. (2021). 5-aminolevulinic acid-mediated plant adaptive responses to abiotic stress. *Plant Cell Reports,**40*, 1451–1469. 10.1007/s00299-021-02690-9 33839877 10.1007/s00299-021-02690-9

[CR45] Secretariat of the Stockholm Convention (SSC). (2019). *All POPs listed in the Stockholm convention*. Retrieved December 5 2024, from http://chm.pops.int/TheConvention/ThePOPs/AllPOPs/tabid/2509/Default.aspx

[CR46] Seo, M., Koiwai, H., Akaba, S., Komano, T., Oritani, T., Kamiya, Y., & Koshiba, T. (2000). Abscisic aldehyde oxidase in leaves of *Arabidopsis thaliana*. *Plant Journal,**23*(4), 481–488. 10.1046/j.1365-313x.2000.00812.x 10.1046/j.1365-313x.2000.00812.x 10972874

[CR47] Sörengård, M., Östblom, E., Köhler, S., & Ahrens, L. (2020). Adsorption behavior of per- and polyfluoroalkyl substances (PFASs) to 44 inorganic and organic sorbents and use of dyes as proxies for PFAS sorption. *Journal of Environmental Chemical Engineering,**8*(3), 103744. 10.1016/j.jece.2020.103744

[CR48] Steenland, K., & Winquist, A. (2021). PFAS and cancer: A scoping review of the epidemiologic evidence. *Environmental Research,**194*, 110690. 10.1016/j.envres.2020.110690 33385391 10.1016/j.envres.2020.110690 PMC7946751

[CR49] Sunderland, E. M., et al. (2019). A review of the pathways of human exposure to poly- and perfluoroalkyl substances (PFASs) and present understanding of health effects. *Journal of Exposure Science & Environmental Epidemiology,**29*(2), 131–147. 10.1038/s41370-019-0100-5 30470793 10.1038/s41370-018-0094-1 PMC6380916

[CR50] Vanommeslaeghe, K., & MacKerell, A. D., Jr. (2012). Automation of the CHARMM General Force Field (CGenFF) I: Bond perception and atom typing. *Journal of Chemical Information and Modeling,**52*, 3144–3154. 23146088 10.1021/ci300363c PMC3528824

[CR51] Wang, M., Maki, C. R., Deng, Y., Tian, Y., & Phillips, T. D. (2017). Development of high-capacity enterosorbents for aflatoxin B1 and other hazardous chemicals. *Chemical Research in Toxicology,**30*(9), 1694–1701. 10.1021/acs.chemrestox.7b00181 28768106 10.1021/acs.chemrestox.7b00154 PMC6684212

[CR52] Wang, M., Orr, A. A., Jakubowski, J. M., Bird, K. E., Casey, C. M., Hearon, S. E., Tamamis, P., & Phillips, T. D. (2021). Enhanced adsorption of per- and polyfluoroalkyl substances (PFAS) by edible, nutrient-amended montmorillonite clays. *Water Research,**188*, 116534. 10.1016/j.watres.2020.116534 33125992 10.1016/j.watres.2020.116534 PMC7725962

[CR53] Washington, J. W., Jenkins, T. M., Rankin, K., & Naile, J. E. (2015). Decades-scale degradation of commercial, side-chain, fluorotelomer-based polymers in soils and water. *Environmental Science & Technology,**49*(2), 915–923. 10.1021/es5051743 25426868 10.1021/es504347u

[CR54] Wee, S. Y., & Aris, A. Z. (2017). Endocrine disrupting compounds in drinking water supply system and human health risk implication. *Environmental International,**106*, 207–233. 10.1016/j.envint.2017.05.022 10.1016/j.envint.2017.05.004 28552550

[CR55] Xu, J., Du, P., Liu, X., Xu, X., Ge, Y., & Zhang, C. (2023a). Curcumin supplementation increases longevity and antioxidant capacity in *Caenorhabditis elegans*. *Frontiers in Pharmacology,**14*, 1195490. 10.3389/fphar.2023.1195490 37346299 10.3389/fphar.2023.1195490 PMC10279890

[CR56] Xu, X., Zhang, C., Xu, X., Cai, R., Guan, Q., Chen, X., Chen, Y., Zhang, Z., Xu Han, Y., Lin, Z., & Lai, Z. (2023b). Riboflavin mediates m6A modification targeted by miR408, promoting early somatic embryogenesis in longan. *Plant Physiology,**192*, 1799–1820. 10.1093/plphys/kiad139 36930572 10.1093/plphys/kiad139 PMC10315286

[CR57] Yu, N., et al. (2015). Distribution of perfluorooctane sulfonate isomers and predicted risk of thyroid hormonal perturbation in drinking water. *Water Research,**76*, 171–180. 10.1016/j.watres.2015.02.053 25813491 10.1016/j.watres.2015.02.047

[CR58] Zhang, W., & Liang, Y. (2022). Performance of different sorbents toward stabilizing per- and polyfluoroalkyl substances (PFAS) in soil. *Environmental Advances,**8*, 100217. 10.1016/j.envadv.2022.100217

[CR59] Zhang, Q., Deng, S., Yu, G., & Huang, J. (2011). Removal of perfluorooctane sulfonate from aqueous solution by crosslinked chitosan beads: Sorption kinetics and uptake mechanism. *Bioresource Technology,**102*(3), 2265–2271. 10.1016/j.biortech.2010.10.051 21044835 10.1016/j.biortech.2010.10.040

[CR60] Zhang, D., Zhang, W., & Liang, Y. (2019). Adsorption of perfluoroalkyl and polyfluoroalkyl substances (PFASs) from aqueous solution—a review. *Science of the Total Environment,**694*, 133606. 10.1016/j.scitotenv.2019.133606 31401505 10.1016/j.scitotenv.2019.133606

[CR61] Zhang, W., Jiang, T., & Liang, Y. (2022). Stabilization of per- and polyfluoroalkyl substances (PFAS) in sewage sludge using different sorbents. *Journal of Hazardous Materials Advances,**6*, 100089. 10.1016/j.hazadv.2022.100089

[CR62] Zhao, L., Bian, J., Zhang, Y., Zhu, L., & Liu, Z. (2014). Comparison of the sorption behaviors and mechanisms of perfluorosulfonates and perfluorocarboxylic acids on three kinds of clay minerals. *Chemosphere,**114*, 51–58. 10.1016/j.chemosphere.2014.07.029 25113183 10.1016/j.chemosphere.2014.03.098

[CR63] Zhou, Q., Deng, S., Yu, Q., Zhang, Q., Yu, G., Huang, J., & He, H. (2010). Sorption of perfluorooctane sulfonate on organo-montmorillonites. *Chemosphere,**78*(6), 688–694. 10.1016/j.chemosphere.2009.11.067 20042218 10.1016/j.chemosphere.2009.12.005

[CR64] Zhou, P., Gu, Q., Zhou, S., & Cui, X. (2024). A novel montmorillonite clay-cetylpyridinium chloride material for reducing PFAS leachability and bioavailability from soils. *Journal of Hazardous Materials,**465*, 133402. 10.1016/j.jhazmat.2023.133402 38183937 10.1016/j.jhazmat.2023.133402

