# Supplementary material for: Adsorption of Per- and Polyfluoroalkyl Substances by Edible Nutraceutical-Amended Montmorillonite Clays: In Vitro, In Vivo and In Silico Enterosorption Strategies
Source: Water Air Soil Pollut. 2025 Apr 4;236(5):293. doi: 10.1007/s11270-025-07930-2 (PMC11971225; doi:10.1007/s11270-025-07930-2)
Supplement: Supplementary file 1 — Supplementary file1 (PDF 4798 KB) [file 11270_2025_7930_MOESM1_ESM.pdf]

Supporting Information

Adsorption of Per- and polyfluoroalkyl substances by edible nutraceutical-amended montmorillonite clays: *in vitro, in vivo* and *in silico* enterosorption strategies

Johnson O. Oladele [a,b], Xenophon Xenophontos [c], Meichen Wang [a,b], Phanourios Tamamis [c,d], Timothy D. Phillips [a,b] *

[a] Interdisciplinary Faculty of Toxicology, Texas A&M University, College Station, TX 77843, USA
[b] Department of Veterinary Physiology and Pharmacology, College of Veterinary Medicine & Biomedical Sciences, Texas A&M University, College Station, TX 77843, USA
[c] Artie McFerrin Department of Chemical Engineering, College of Engineering, Texas A&M University, College Station, TX 77843, USA
[d] Department of Materials Science and Engineering, College of Engineering, Texas A&M University, College Station, TX 77843, USA

*Corresponding author: tphillips@cvm.tamu.edu

Computational Methodology

Firstly, we determined the protonation state of caffeine, curcumin and riboflavin at pH 2 and pH 7. According to these studies (Khan & Mohan, 1973; Quick et al., 2013) riboflavin has a positive (+1) charge at pH 2, with the two studies showing the exact position of the protonated group. For caffeine's and curcumin's protonation state we used graph-convolutional neural network model-based calculations by MolGpKa (Pan et al., 2021), in line with their protonation state at pH 2 based on their pK_a from literature (caffeine (Wang et al., 2022), curcumin (Martínez-Guerra et al., 2019)). Caffeine has a positive (+1) charge at pH 2, while curcumin has no charge at pH 2. The protonation state of these compounds was determined at pH 7 using the same methods. All three compounds have no charge at pH 7. Then we determined the protonation state of the four PFAS species we chose to investigate in mixtures: GenX, PFOA, PFOS, and PFBS, at pH 2 and pH 7. All four PFAS species were investigated at their monoanionic states (charge -1) at both pH conditions, after calculations done using MolGpKa (Pan et al., 2021) showed pK_a values which were much lower than 2; i.e., 0.0 for GenX, 0.1 for PFOA, -2.9 for both PFOS and PFBS. For PFOS and PFBS, pK_a values much lower than 2 were also reported in literature (Lasáková & Jandera, 2019; Steinle-Darling & Reinhard, 2008). Additionally, a recent study for PFOA (Vierke et al., 2013) reported a pK_a value of 0.5, which supported the MolGpKa (Pan et al., 2021)

calculations and the investigation of the monoanionic state. These compounds with their respective protonation state at pH 2 and pH 7 are shown in Table S1.

The initial structures for the simulations of uncharged caffeine, or curcumin, or riboflavin were taken from PubChem. The initial structures of the positively charged caffeine and riboflavin were constructed using CHARMM-GUI Ligand Reader and Modeler (Jo et al., 2008; Brooks et al., 2009; Lee et al., 2016; Kim et al., 2017), starting from their corresponding uncharged structures from PubChem and adding a hydrogen to the group according to MolGpKa (Pan et al., 2021) for caffeine, and the previously mentioned studies for riboflavin (Khan & Mohan, 1973; Quick et al., 2013). The topology and parameter files for all compounds in all corresponding states were obtained using CGenFF (Vanommeslaeghe & MacKerell, 2012) version 3.0. To construct the initial structure of the monoanionic states of the PFAS molecules, CHARMM-GUI Ligand Reader and Modeler (Jo et al., 2008; Brooks et al., 2009; Lee et al., 2016; Kim et al., 2017) was used, starting from their corresponding uncharged structures from PubChem and removing the hydrogen from their functional groups. Afterwards, the topology and parameter files were obtained through CGenFF via CHARMM-GUI (Jo et al., 2008; Brooks et al., 2009; Lee et al., 2016). As in our previous studies (Orr et al., 2020; Orr et al., 2021), the initial clay structure at acidic conditions was constructed using CHARMM-GUI (Jo et al., 2008; Brooks et al., 2009; Lee et al., 2016; Kim et al., 2017; Choi et al., 2022; Heinz et al., 2013). A model of two layers of clay with chemical composition $(Si_4)^{IV}(Al_{1.67}Mg_{0.33})^{VI}O_{10}(OH)_2$, dimension 50x50 Å^2, miller indices 001, and a ratio of defect of 0.33333 was obtained. The two layers were subsequently separated at a distance of 21 Å (Greenland & Quirk, 1960) and the ions from the surfaces were removed and were later introduced randomly in the water box where they were allowed to move freely. To construct the initial structure for clay at neutral conditions, we started from the acidic structure as described above and we removed specific hydrogens and hydroxyl groups from the clay edges, based on differences between acidic and neutral calcium montmorillonite model of INTERFACE FF (Heinz et al., 2013). The INTERFACE FF (Heinz et al., 2013) force field was used for the parameterization of clay, in conjunction with CHARMM-GUI (Jo et al., 2008; Brooks et al., 2009; Lee et al., 2016).

Initially, we investigated the ability of caffeine, curcumin and riboflavin to be amended on clay at acidic conditions. We employed 100 ns molecular dynamics simulations in explicit solvent, for each amendment compound independently. For each amendment compound, 24 copies of the compound in the acidic state, produced as described above, were simulated in complex with the clay. Input files to set up and simulate the compound-clay systems were initially generated using CHARMM-GUI (Jo et al., 2008; Brooks et al., 2009; Lee et al., 2016; Kim et al., 2017; Choi et al., 2022; Heinz et al., 2013) and were later modified. Initially, the two clay layers were centered in a cubic (120 Å) periodic boundary conditions box and solvated by explicit water molecules in CHARMM (Brooks et al., 2009). The sodium ions provided by default by CHARMM-GUI (Jo et al., 2008; Brooks et al., 2009; Lee et al., 2016; Kim et al., 2017; Choi et al., 2022; Heinz et al., 2013) for the clay model were manually deleted and were replaced with calcium ions which were

placed randomly and allowed to freely move in the water box, to neutralize the constructed systems. The 24 copies of the amendment in each case were randomly placed and oriented close to the box, no more than ~35 Å away from the clay and 5 Å or further away from any atom of the clay or other amendment copies. Before the production, a 200 ps equilibration (NVT) run at 300 K was performed. Subsequently, an 80 ns production (NPT) run was performed at 300 K, 1 atm and using an isotropic barostat. During both the equilibration and the production steps magnesium and aluminum atoms of clay were constrained with a force constant of 400 kJ/(mol nm^2). Both equilibration and production steps were performed using OpenMM (Eastman et al., 2017). The final snapshot of each simulation of the amendment compounds with the clay were extracted and were used as the initial structure of the amended clays for later simulations.

Subsequently, we investigated the ability of the three amended clays to bind PFAS molecules from PFAS mixture at neutral conditions. We employed triplicates of 200 ns molecular dynamics simulations in explicit solvent, for each amended clay, independently. In each case, 24 copies of PFAS molecules (6 copies of each one of the four PFAS species) in their monoanionic state, produced as described above, were simulated in complex with the amended clay. The structure corresponding to the last simulation snapshot of the amending process as described above was used to extract the initial coordinates of the clay and the amendments, however both the clay and the amendments were simulated in their corresponding states at pH 7. Input files to set up and simulate the amended clay with the PFAS mixture were initially generated using CHARMM-GUI (Jo et al., 2008; Brooks et al., 2009; Lee et al., 2016; Kim et al., 2017; Choi et al., 2022; Heinz et al., 2013), and were later modified. Initially, the amended clay was adjusted in a cubic (120 Å) periodic boundary conditions box, such that all amendment molecules exist within the box and are solvated by explicit water molecules in CHARMM (Brooks et al, 2009). The 24 copies of PFAS molecules in each case were randomly placed and oriented in the box, such that they would have a distance of 10 Å or larger with other PFAS molecules, or with the amendments, or with the clay. Before the production, a 200 ps equilibration (NVT) run at 300 K was performed. Subsequently, a 200 ns production (NPT) run was performed at 300 K, 1 atm and using an isotropic barostat. During both the equilibration and the production steps magnesium and aluminum atoms of clay were constrained with a force constant of 400 kJ/(mol nm^2). Both equilibration and production steps were performed using OpenMM (Eastman et al., 2017). Using the same protocol as described above we have also employed triplicate 200 ns molecular dynamics simulations in explicit solvent, for the PFAS mixture and the parent (unamended) clay. We refer to these as the control runs.

After the completion of all simulations, in-house FORTRAN programs were utilized to analyze the simulation trajectories and quantify the binding capacity of the PFAS molecules from the mixture to the parent and amended clays. Simulations were analyzed with snapshots extracted every 1 ns, thereby 200 snapshots were analyzed per simulation. We determined the binding percentage probability of the PFAS mixture to parent clay and to each one of the amended clays. The binding percentage probability was calculated by counting the total number of interactions of each PFAS molecule of any species with the parent or amended clay and it was normalized by the

total number of copies of PFAS molecules (24) in the simulations and the total number of snapshots analyzed (200). The average binding percentage probability of the PFAS mixture to parent clay and to each one of the amended clays was calculated over the triplicate runs. The contribution of each PFAS species to the average binding percentage probability was also calculated.

In addition, using our in-house FORTRAN programs we explained with atomistic details the interactions between the PFAS molecules and the parent and amended clays. More specifically, we studied (I) interactions of the amendments with parent clay, (II) interactions of the PFAS with the amended as well as parent clays. To do that, we decomposed each PFAS and each amendment into chemical groups, as shown in Figure S1 and S2, respectively. Subsequently, we decomposed interactions into: (ai) direct-assisted interactions, which are interactions of PFAS with the clay and amendment molecules (bound to the clay) simultaneously; (aii) direct-helped interactions, which are interactions of PFAS with the clay and amendment molecules (not bound to clay), simultaneously; and (aiii) indirect-assisted interactions, which are interactions of PFAS only with amendment molecules which are either bound directly to clay or they are part of a cluster of amendment molecules indirectly bound to clay. PFAS molecules that were part of PFAS aggregates of which at least one molecule was interacting with the amendment molecules via (ai)-(aiii) were also counted. In few cases, clusters of PFAS molecules were identified bound to the clays, and in these cases, only the directly bound PFAS molecule was considered as part of the direct interactions. Snapshots of simulations were obtained and visualized using VMD (Humphrey et al., 1996).

Figures and Tables

Table S1: The table lists the amendments and the PFAS species that were investigated, with their corresponding PubChem ID and their charge at pH 2 and pH 7.

PFAS	PubChem CID	Charge at pH 2	Charge at pH 7
Caffeine	2519	+1	0
Curcumin	969516	0	0
Riboflavin	493570	+1	0
PFOA (Perfluorooctanoic acid)	9554	-1	-1
PFOS (Perfluorooctane sulfonic acid)	74483	-1	-1
PFBS (Perfluorobutane sulfonic acid)	67815	-1	-1
GenX (Perfluoro-2-methyl-3-oxahexanoic acid)	114481	-1	-1

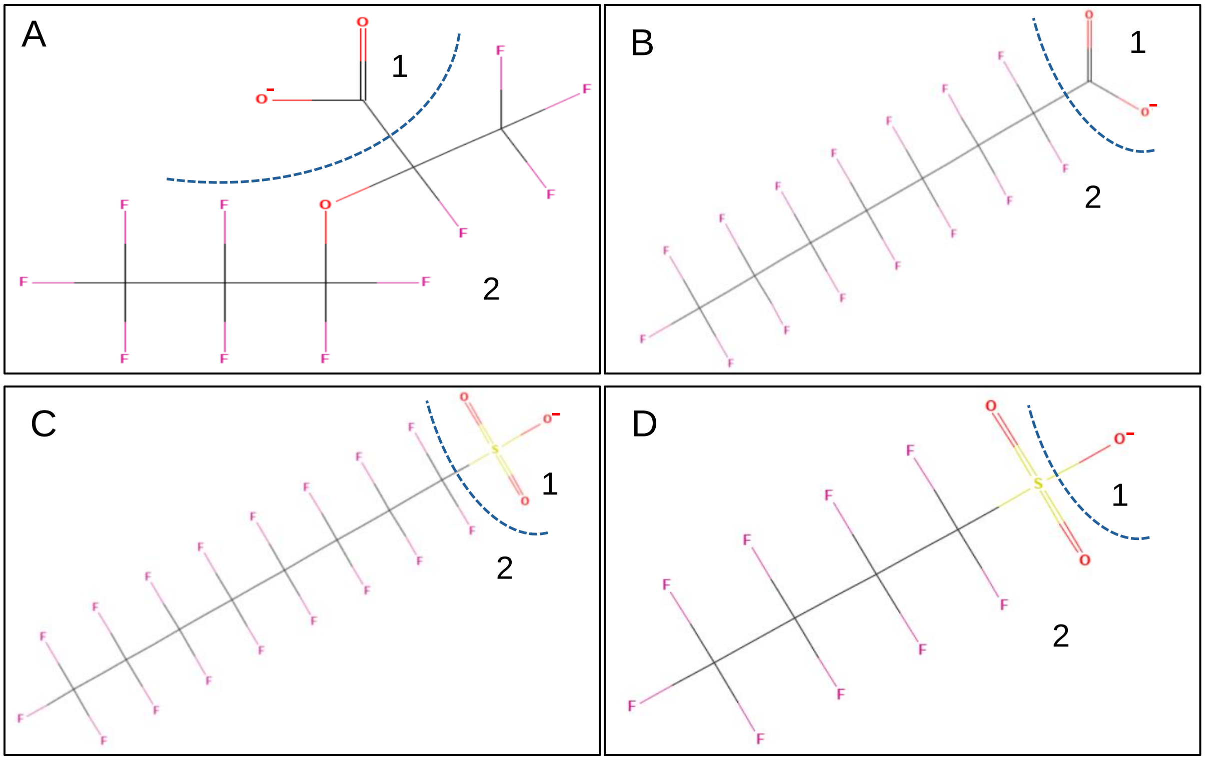

Figure S1: The structures of the PFAS molecules investigated, and their decomposition into chemical groups. Panels A-D represent GenX, PFOA, PFOS, and PFBS, respectively. The blue dashed line shows the decomposition into chemical groups for each molecule. Chemical groups 1 represent the functional groups of the PFAS molecules, while chemical groups 2 represent the perfluoroalkyl tail of the PFAS molecules.

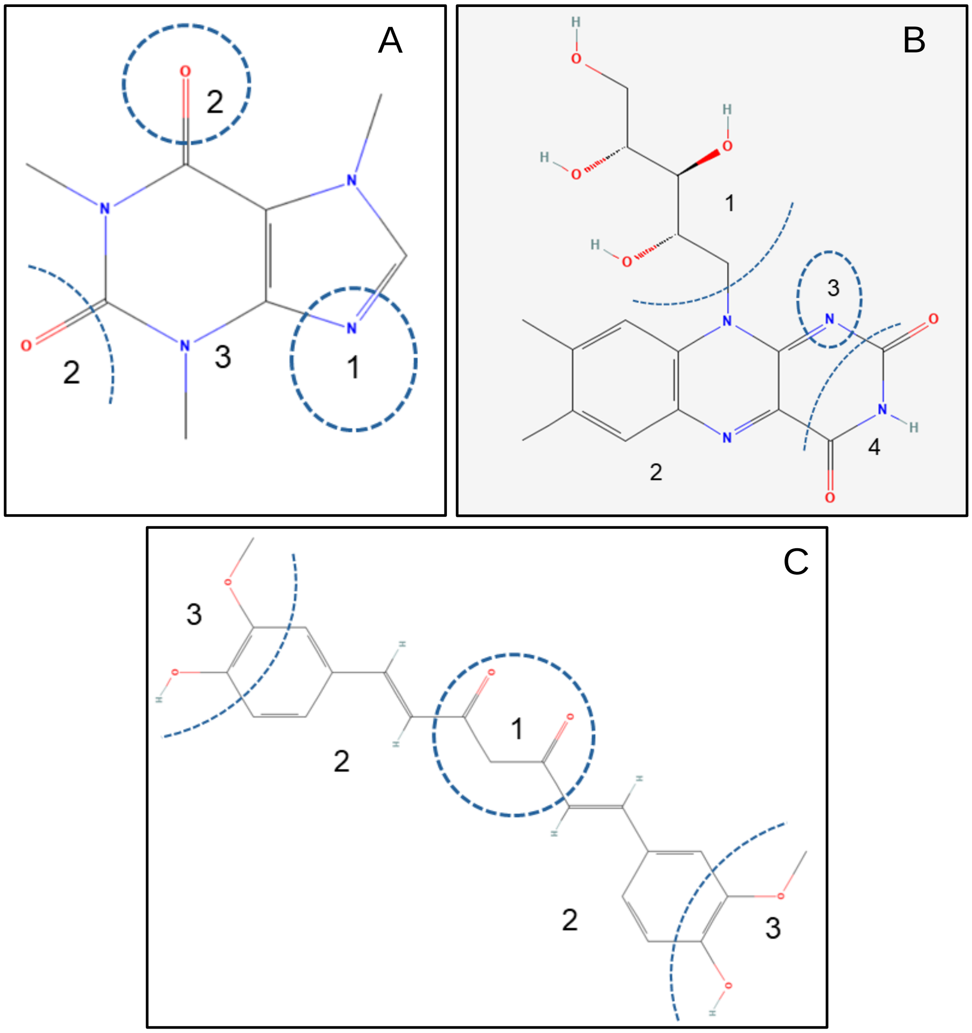

Figure S2: The structures of the three amendments we studied at pH 7, and their decomposition into chemical groups. Panels A-C represent caffeine, riboflavin, and curcumin, respectively. The blue dashed line shows the decomposition into chemical groups for each molecule. For caffeine (Panel A), groups 1 and 2 correspond to oxygen and nitrogen atoms that can be hydrogen-bond acceptors and group 3 corresponds to the non-polar groups (methyl groups and rings). For riboflavin (Panel B), groups 1, 3 and 4 correspond to polar moieties. Group 1 is an alkyl tail with four hydroxyl groups. Group 2 corresponds to the non-polar part of riboflavin, comprised of methyl groups and rings. Groups 3 and 4 correspond to oxygen and nitrogen atoms that can be hydrogen bond acceptors. For curcumin (Panel C), group 1 corresponds to oxygen atoms in the middle of the molecule that can be hydrogen bond acceptors. Groups 2 correspond to the non-polar part of curcumin. Groups 3 correspond to the polar groups at the edges. The hydroxyl groups can be both donors and acceptors in hydrogen bonds, while the ether oxygens can only be a hydrogen bond acceptor.

References

1. Khan, M. M. T., & Mohan, M. S. (1973). The metal chelates of riboflavin and riboflavin monophosphate. *Journal of Inorganic and Nuclear Chemistry, 35*(5), 1749-1755. https://doi.org/10.1016/0022-1902(73)80275-1
2. Quick, M., Weigel, A., & Ernsting, N. P. (2013). Fluorescence following excited-state protonation of riboflavin at N(5). *The Journal of Physical Chemistry B, 117*(18), 5441-5447. https://doi.org/10.1021/jp312571d
3. Pan, X., Wang, H., Li, C., Zhang, J. Z. H., & Ji, C. (2021). MolGpka: A Web Server for Small Molecule p*K*a Prediction Using a Graph-Convolutional Neural Network. *Journal of chemical information and modeling*, *61*(7), 3159–3165. https://doi.org/10.1021/acs.jcim.1c00075
4. Wang, X., Zhang, H., Wei, Y., Bao, L., Liu, S., Yuan, S., & Yuan, S. (2022). Effect of pH on caffeine removal from aqueous media by graphene/graphene oxide adsorption. *Colloids and Surfaces A: Physicochemical and Engineering Aspects*, 644, 128864. https://doi.org/10.1016/j.colsurfa.2022.128864
5. Martínez-Guerra, J., Palomar-Pardavé, M., Romero-Romo, M., Corona-Avendaño, S., Rojas-Hernández, A., & Ramírez-Silva, M. T. (2019). New insights on the chemical stability of curcumin in aqueous media at different pH: Influence of the experimental conditions. *International Journal of Electrochemical Science, 14*(6), 5373-5385. https://doi.org/10.20964/2019.06.24
6. Lasáková, M., & Jandera, P. (2009). Molecularly imprinted polymers and their application in solid phase extraction. *Journal of separation science*, *32*(5-6), 799–812. https://doi.org/10.1002/jssc.200800506
7. Steinle-Darling, E., & Reinhard, M. (2008). Nanofiltration for trace organic contaminant removal: Structure, solution, and membrane fouling effects on the rejection of perfluorochemicals. *Environmental Science & Technology, 42*(14), 5292-5297. https://doi.org/10.1021/es703207s
8. Vierke, L., Berger, U., & Cousins, I. T. (2013). Estimation of the acid dissociation constant of perfluoroalkyl carboxylic acids through an experimental investigation of their water-to-air transport. *Environmental Science & Technology, 47*(19), 11032-11039. https://doi.org/10.1021/es402691z
9. S. Jo, T. Kim, V.G. Iyer, and W. Im (2008). CHARMM-GUI: A Web-based Graphical User Interface for CHARMM. *J. Comput. Chem. 29:1859-1865*
10. Brooks, B. R., Brooks, C. L., 3rd, Mackerell, A. D., Jr, Nilsson, L., Petrella, R. J., Roux, B., Won, Y., Archontis, G., Bartels, C., Boresch, S., Caflisch, A., Caves, L., Cui, Q., Dinner, A. R., Feig, M., Fischer, S., Gao, J., Hodoscek, M., Im, W., Kuczera, K., … Karplus, M. (2009). CHARMM: the biomolecular simulation program. *Journal of computational chemistry*, *30*(10), 1545–1614. https://doi.org/10.1002/jcc.21287
11. Lee, J., Cheng, X., Swails, J. M., Yeom, M. S., Eastman, P. K., Lemkul, J. A., Wei, S., Buckner, J., Jeong, J. C., Qi, Y., Jo, S., Pande, V. S., Case, D. A., Brooks, C. L., 3rd, MacKerell, A. D., Jr, Klauda, J. B., & Im, W. (2016). CHARMM-GUI Input Generator for NAMD, GROMACS, AMBER, OpenMM, and CHARMM/OpenMM Simulations Using the CHARMM36 Additive Force Field. *Journal of chemical theory and computation*, *12*(1), 405–413. https://doi.org/10.1021/acs.jctc.5b00935

12. S. Kim, J. Lee, S. Jo, C.L. Brooks III, H.S. Lee, and W. Im (2017) CHARMM-GUI Ligand Reader and Modeler for CHARMM Force Field Generation of Small Molecules. *J. Comput. Chem. 38:1879-1886*
13. Vanommeslaeghe, K., & MacKerell, A. D., Jr (2012). Automation of the CHARMM General Force Field (CGenFF) I: bond perception and atom typing. *Journal of chemical information and modeling*, *52*(12), 3144–3154. https://doi.org/10.1021/ci300363c
14. Orr, A. A., He, S., Wang, M., Goodall, A., Hearon, S. E., Phillips, T. D., & Tamamis, P. (2020). Insights into the interactions of bisphenol and phthalate compounds with unamended and carnitine-amended montmorillonite clays. *Computers & chemical engineering*, *143*, 107063. https://doi.org/10.1016/j.compchemeng.2020.107063
15. Orr, A. A., Wang, M., Beykal, B., Ganesh, H. S., Hearon, S. E., Pistikopoulos, E. N., Phillips, T. D., & Tamamis, P. (2021). Combining Experimental Isotherms, Minimalistic Simulations, and a Model to Understand and Predict Chemical Adsorption onto Montmorillonite Clays. *ACS omega*, *6*(22), 14090–14103. https://doi.org/10.1021/acsomega.1c00481
16. Choi, Y. K., Kern, N. R., Kim, S., Kanhaiya, K., Afshar, Y., Jeon, S. H., Jo, S., Brooks, B. R., Lee, J., Tadmor, E. B., Heinz, H., & Im, W. (2022). CHARMM-GUI Nanomaterial Modeler for Modeling and Simulation of Nanomaterial Systems. *Journal of chemical theory and computation*, *18*(1), 479–493. https://doi.org/10.1021/acs.jctc.1c00996
17. Heinz, H., Lin, T. J., Mishra, R. K., & Emami, F. S. (2013). Thermodynamically consistent force fields for the assembly of inorganic, organic, and biological nanostructures: the INTERFACE force field. *Langmuir : the ACS journal of surfaces and colloids*, *29*(6), 1754–1765. https://doi.org/10.1021/la3038846
18. Greenland, D. J., & Quirk, J. P. (1960). Adsorption of 1-n-Alkyl Pyridinium Bromides by Montmorillonite. *Clays and Clay Minerals (National Conference on Clays and Clay Minerals)*, *9*, 484–499. https://doi.org/10.1346/CCMN.1960.0090136
19. Eastman, P., Swails, J., Chodera, J. D., McGibbon, R. T., Zhao, Y., Beauchamp, K. A., Wang, L. P., Simmonett, A. C., Harrigan, M. P., Stern, C. D., Wiewiora, R. P., Brooks, B. R., & Pande, V. S. (2017). OpenMM 7: Rapid development of high performance algorithms for molecular dynamics. *PLoS computational biology*, *13*(7), e1005659. https://doi.org/10.1371/journal.pcbi.1005659
20. Humphrey, W., Dalke, A., & Schulten, K. (1996). VMD: visual molecular dynamics. *Journal of molecular graphics*, *14*(1), 33–28. https://doi.org/10.1016/0263-7855(96)00018-5
